# Extending the Tavis–Cummings model for molecular ensembles—Exploring the effects of dipole self-energies and static dipole moments

**DOI:** 10.1063/5.0214362

**Published:** 2024-07-28

**Authors:** Lucas Borges, Thomas Schnappinger, Markus Kowalewski

**Affiliations:** Department of Physics, Stockholm University, AlbaNova University Center, SE-106 91 Stockholm, Sweden

## Abstract

Strong coupling of organic molecules to the vacuum field of a nanoscale cavity can be used to modify their chemical and physical properties. We extend the Tavis–Cummings model for molecular ensembles and show that the often neglected interaction terms arising from the static dipole moment and the dipole self-energy are essential for a correct description of the light–matter interaction in polaritonic chemistry. On the basis of a full quantum description, we simulate the excited-state dynamics and spectroscopy of MgH^+^ molecules resonantly coupled to an optical cavity. We show that the inclusion of static dipole moments and the dipole self-energy is necessary to obtain a consistent model. We construct an efficient two-level system approach that reproduces the main features of the real molecular system and may be used to simulate larger molecular ensembles.

## I Introduction

Polaritonic chemistry, exploring chemical reactions strongly coupled to a confined electromagnetic field, is an emerging field of research at the interface between quantum optics, quantum chemistry, and materials science.^1–3^ By coupling molecules to confined light modes in an optical cavity, the interplay of local excitations and collective excitations in ensembles of quantum emitters gives rise to hybrid states of light–matter known as polaritons.^4–7^ Depending on whether the quantized cavity modes are coupled via their characteristic frequencies to electronic or vibrational degrees of freedom of molecules, the situation is described as electronic-strong coupling (ESC) or vibrational-strong coupling (VSC), respectively. Under ESC, it becomes possible to modify the photochemistry/photophysics of molecules, including photoinduced reactions and electronic spectroscopy.^8–17^

The observed effects of molecular ESC and VSC are often discussed phenomenologically by adapting models such as the Rabi model,^18^ the Dicke model,^19^ the Jaynes–Cummings (JC) model,^20^ or the Tavis–Cummings (TC) model.^21^ However, all of these models were developed to describe single atoms or atomic ensembles represented by a two-level system (TLS). The addition of nuclear degrees of freedom to the JC model and the TC model makes it possible to describe processes in the presence of ESC as non-adiabatic processes.^17,22,23^ These models are also used to simulate large molecular ensemble sizes due to their simplified description of the coupling and molecules.^12,24–28^ Moreover, it has been demonstrated that the concept of non-adiabatic transitions can even be applied to VSC.^29,30^

However, most of these models do not take into account static dipole moments or the feedback of the light field on the electronic structure. It has been demonstrated that both static dipoles and a self-consistent treatment of the electronic structure in the presence of the photon field can be crucial for the description of polaritonic chemistry.^31–37^ In addition, the dipole self-energy (DSE) gives rise to a cavity-induced interaction between molecules in an ensemble and depends on the relative molecular orientation of the ensemble.^33,34,37,38^ In recent years, established electronic structure methods have been generalized to include the effects of quantum light–matter interactions^34,39,40^ and used to determine the polaritonic states of molecule–cavity hybrid systems based on the full non-relativistic Pauli–Fierz Hamiltonian.^31,35,41,42^ These *ab initio* methods are more accurate, but because of their computational cost, they are limited to single molecules and small molecular ensembles.

In this paper, we build on the framework of the molecular TC model to include both static dipole moments and DSE contributions while using only field-free molecular properties. The starting point is the non-relativistic Pauli–Fierz Hamiltonian in the length gauge. However, since we want to study systems with static dipole moments, the separation into “matter” and “photon” degrees of freedom is no longer trivial.^43,44^ We discuss how this ambiguity between light and matter can be partially circumvented by a coherent state (CS) transformation.^39,43,45,46^ Based on this CS Hamiltonian, we derive a generalized TC Hamiltonian for a molecular system coupled to a single-cavity mode under ESC conditions.

As a first test case for the generalized molecular TC Hamiltonian, we simulate MgH^+^ molecules resonantly coupled to an optical cavity. We investigate the influence of static dipole moments and the influence of the DSE on the dynamics and compare the results with the standard TC Hamiltonian. Moreover, we analyze the effect of the CS transformation that becomes necessary when the molecular ensemble has a nonzero total dipole moment. In the second step, we calculate and discuss the polaritonic absorption spectra of coupled MgH^+^-cavity systems. On the basis of these results, we construct an effective two-level system (TLS) model for larger ensembles of MgH^+^ molecules. After optimizing the TLS parameters, we analyze the structure of this reduced Hamiltonian and study the collective effects induced by the interaction with the cavity mode.

## II Theory And Models

In the following, we make use of the non-relativistic Pauli–Fierz Hamiltonian in the length gauge representation^31,35,41,42^ to describe the interaction of molecules with the confined electromagnetic field. Atomic units (*ħ* = 4*πε*_0_ = *m*_*e*_ =1) are used throughout the paper unless otherwise noted, and bold symbols denote vectors.

The corresponding Pauli–Fierz Hamiltonian *Ĥ*_PF_ for a single cavity mode within the Born–Oppenheimer approximation (BOA) takes the form

(1) $$\begin{array}{l} {\hat{H}}_{\text{PF}}={\hat{T}}_{nuc}+{\hat{H}}_{el}+\omega_{c}({\hat{a}}^{†}\hat{a}+\frac{1}{2})\\ \;\;\;\;\;\;\;\;\;\;\;\;\;\;\;-\sqrt{\frac{\omega_{c}}{2}}({\hat{a}}^{†}+\hat{a})(\lambda \cdot \hat{\mu })+\frac{1}{2}{\left(\lambda .\hat{\mu }\right)}^{2}, \end{array}$$

where ${\hat{T}}_{nuc}$ is the nuclear kinetic energy operator and *Ĥ*_*el*_ is the electronic Hamiltonian, both defining the Hamiltonian of the molecular subsystem *Ĥ*_*m*_. The third term in Eq. (1) is purely photonic and describes the single-cavity mode as a quantum harmonic oscillator with a frequency of *ω*_*c*_. The operators *â*^†^ and *â* are the bosonic photon creation and annihilation operators.^47^ The fourth term describes the dipole coupling of the photon mode and molecular degrees of freedom, where $\hat{\mu }$ is the molecular dipole moment operator and

(2) $$\lambda =e\lambda =e\sqrt{\frac{4\pi }{V_{c}}}$$

is the coupling parameter. Here, *V*_*c*_ is the cavity quantization volume, and ***e*** is the polarization vector of the photon mode. The last term in Eq. (1) is the DSE contribution,^31,32,48^ which describes the self-polarization of the molecule–cavity system.

To compare the dynamics of different ensembles with a varying number of molecules, we keep the collective coupling strength *λ*_*c*_ constant by scaling the single molecule coupling strength ***λ*** with $1/\sqrt{N_{mol}}$,

(3) $$\lambda =\frac{\lambda_{c}}{\sqrt{N_{mol\;}}}e,$$

where *N*_*mol*_ is the number of molecules, and *λ*_*c*_ is then treated as a tunable coupling parameter.

### A The coherent state transformation

The transformation of the Pauli–Fierz Hamiltonian to the dipole gauge leads to a mixing of the light and matter degrees of freedom.^43,44^ The consequence is a shift of the photon mode, which arises for molecular ensembles with a static dipole moment. In Eq. (1), the photonic part and the light–matter interaction are written in terms of the photonic creation and annihilation operators *â*^†^ and *â*, which are defined for an empty cavity mode. To visualize how *â* changes for a system with a static dipole moment, we express the photon mode in terms of photon displacement coordinates *q*_*c*_ and *p*_*c*_,^47,49^

(4) $$q_{c}=\frac{1}{\sqrt{2\omega_{c}}}\left(\hat{a}+{\hat{a}}^{†}\right),\;p_{c}=-i\sqrt{\frac{\omega_{c}}{2}}\left(\hat{a}-{\hat{a}}^{†}\right).$$

**Fig. 1:**
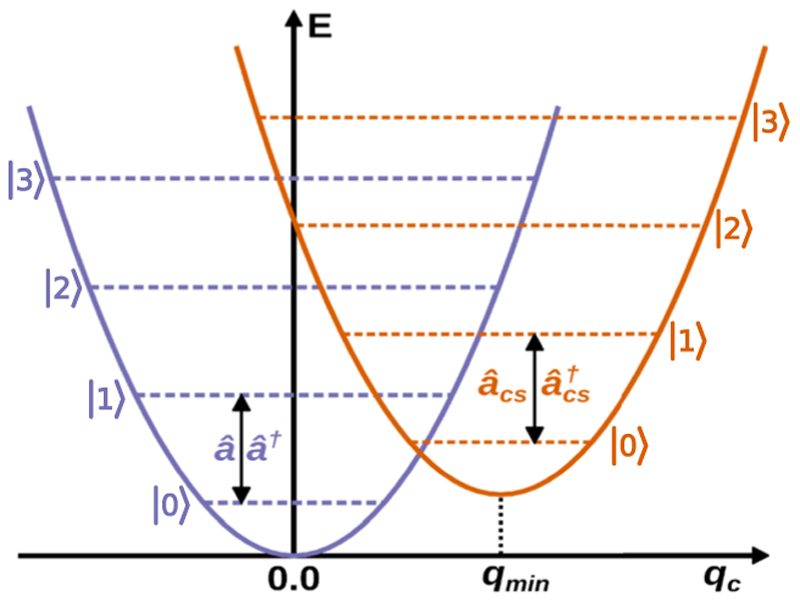
Schematic representation of the harmonic potential for an uncoupled cavity mode (purple) and a cavity mode coupled to a molecular system with a static dipole moment (orange). Due to the light–matter interaction, the potential is shifted in energy, as is the displacement field coordinate *q*_*c*_. Photonic eigenstates are indicated by colored dashed lines, and ∣0⟩ → ∣1⟩ transitions are marked with the corresponding creation and annihilation operators.

The photon mode potentials for a coupled (orange) and an uncoupled (purple) case are shown in Fig. 1. For the uncoupled cavity mode (purple in Fig. 1), the minimum of the harmonic potential is at *q*_*c*_ = 0. The corresponding creation and annihilation operators *â*^†^ and *â* are the usual ladder operators of the quantum harmonic oscillator. The coupling of a molecular system with a static dipole moment affects both the molecule and the cavity mode. The light–matter interaction shifts the photon mode potential in *q*_*c*_ (Fig. 1, orange). The minimum of the shifted harmonic potential for a given nuclear configuration ***R*** is at

(5) $$q_{\text{min}}(R)=-\frac{\lambda \cdot {\langle \hat{\mu }\rangle }_{0}}{\omega_{c}},$$

with ${\langle \hat{\mu }\rangle }_{0}\equiv {\langle \hat{\mu }\rangle }_{0}(R)$ being the static dipole moment function of the molecular ground state.^34,50^ It becomes clear that *â*^†^ and *â* are no longer valid ladder operators for the shifted cavity field potential. The same holds for the number operator $\hat{N}={\hat{a}}^{†}\hat{a}$, which no longer produces valid photon numbers. Note that the CS transformation becomes relevant as soon as the ensemble exhibits a static dipole moment. For a more detailed discussion of this topic, we refer the reader to Refs. 4, 33, 34, and 44.

To compensate for the shift in the photon mode, the coherent state transformation is used.^39,43,45,46^ The unitary transformation

(6) $${\hat{U}}_{cs}(R)=e^{z\left({\hat{a}}^{†}-\hat{a}\right)}\;\text{with}\;z(R)=q_{\text{min}}\sqrt{\frac{{\text{ω}}_{c}}{2}}=-\frac{\text{λ}\cdot {\langle \hat{\mu }\rangle }_{0}}{\sqrt{2{\text{ω}}_{c}}}$$

yields new annihilation and creation operators ${\hat{a}}_{cs}^{†}(R)$ and *â*_*cs*_ (***R***), which now depend on the nuclear configuration through the static dipole moments. These operators can be expressed in terms of the original operators *â*^†^ and *â*,

(7) $$\begin{array}{l} {\hat{a}}_{cs}^{†}(R)={\hat{U}}_{cs}{\hat{a}}^{†}{\hat{U}}_{cs}^{†}={\hat{a}}^{†}-z={\hat{a}}^{†}+\frac{\lambda \cdot {\langle \hat{\mu }\rangle }_{0}(R)}{\sqrt{2\omega_{c}}},\\ \;\;\;\;{\hat{a}}_{cs}(R)={\hat{U}}_{cs}\hat{a}{\hat{U}}_{cs}^{†}=\hat{a}-z=\hat{a}+\frac{\lambda \cdot {\langle \hat{\mu }\rangle }_{0}(R)}{\sqrt{2\omega_{c}}}. \end{array}$$

Applying the same CS transformation to the full Pauli–Fierz Hamiltonian *Ĥ*_PF_ yields the corresponding operators in the CS basis,

(8) $$\begin{array}{l} {\hat{H}}_{cs}={\hat{U}}_{cs}{\hat{H}}_{\text{PF}}{\hat{U}}_{cs}^{†}={\hat{H}}_{m}+\omega_{c}({\hat{a}}^{†}\hat{a}+\frac{1}{2})\\ \;\;\;\;\;\;\;\;\;\;\;-\sqrt{\frac{\omega_{c}}{2}}({\hat{a}}^{†}+\hat{a})(\lambda \cdot \tilde{\mu })+\frac{1}{2}{(}^{\lambda \cdot \tilde{\mu })2}, \end{array}$$

with $\tilde{\mu }=\hat{\mu }-{\langle \hat{\mu }\rangle }_{0}$ describing the change in dipole moment with respect to the ground state. As a consequence, the DSE contribution takes the following form:

$$\frac{1}{2}{\left(\lambda \cdot \tilde{\mu }\right)}^{2}=\frac{1}{2}{\left(\lambda \cdot \hat{\mu }\right)}^{2}-(\lambda \cdot \hat{\mu })\left(\lambda \cdot {\langle \hat{\mu }\rangle }_{0}\right)+\frac{1}{2}{\left(\lambda \cdot {\langle \hat{\mu }\rangle }_{0}\right)}^{2}.$$

In the same way, $\hat{N}$ can also be transformed into the CS basis.

In the following, we will use the Pauli–Fierz Hamiltonian in the CS basis ${\hat{H}}_{cs}$ to describe the cavity–molecule systems, unless otherwise noted. Assuming that the dipole moment is oriented in parallel to the polarization axis of the cavity mode, the scalar product $\text{λ}\cdot \tilde{\mu }$ is reduced to the simple product $\lambda \tilde{\mu }$. The Hamiltonians shown in Eqs. (1) and (8) are formally equivalent in the complete basis limit.^46,51^ However, the photonic states described by *â*^†^ and *â* are not necessarily proper annihilation or creation operators of the coupled cavity–molecule system. Note that we use Eq. (8) with the BOA applied throughout the rest of the paper. All operators are then operators that act on the electronic eigenstates, the nuclear coordinates, and the Fock-states of the photon field.

### B The extended Tavis–Cummings Hamiltonian

In this paper, we do not solve the coupled electron–polariton part of ${\hat{H}}_{\text{CS}}$ self-consistently but use field-free molecular properties, such as potential energy surfaces (PESs) and dipole moment functions, in an adiabatic basis, assuming the BOA, in the derivation of the generalized Tavis–Cummings Hamiltonian. The molecular TC model was formulated under the assumption that the interacting molecules do not have a static dipole moment. In the following, we extend the molecular TC model to include static dipole moments and the DSE terms of Eq. (1). We will refer to this generalized model as the molecular extended Tavis–Cummings (ETC) ansatz.

The molecular Hamiltonian ${\hat{H}}_{m}^{\left(i\right)}$ of the *i*th molecule has two electronic states: a ground state *g* and the first excited state *e*,

(10) $${\hat{H}}_{m}^{\left(i\right)}={\hat{T}}_{nuc}+V_{g}\left(R_{i}\right){\hat{\sigma }}^{\left(i\right)}{\hat{\sigma }}^{(i)†}+V_{e}\left(R_{i}\right){\hat{\sigma }}^{(i)†}{\hat{\sigma }}^{\left(i\right)},$$

where ***R***_*i*_ is a set of nuclear coordinates of the *i*th molecule. The operators ${\hat{\sigma }}^{\left(i\right)}=|g_{i}\rangle \langle e_{i}|$ and ${\hat{\sigma }}^{{\left(i\right)}^{†}}=|e_{i}\rangle \langle g_{i}|$ annihilate and create, respectively, an excitation in the electronic subspace on the *i*th molecule, defined by the ground and first excited state PESs *V*_*g*_ (***R***) and *V*_*e*_ (***R***), respectively. The corresponding dipole moment and squared dipole moment operators for the individual molecule of the *i*th molecule can be expressed as follows:

(11) $${\hat{\mu }}^{\left(i\right)}=\mu_{gg}{\hat{\sigma }}^{\left(i\right)}{\hat{\sigma }}^{(i)†}+\mu_{ee}{\hat{\sigma }}^{(i)†}{\hat{\sigma }}^{\left(i\right)}+\mu_{eg}\left({\hat{\sigma }}^{\left(i\right)}+{\hat{\sigma }}^{(i)†}\right),$$

(12) $${\left({\hat{\mu }}^{\left(i\right)}\right)}^{2}=\mu_{gg}^{2}{\hat{\sigma }}^{\left(i\right)}{\hat{\sigma }}^{(i)†}+\mu_{ee}^{2}{\hat{\sigma }}^{(i)†}{\hat{\sigma }}^{\left(i\right)}+\mu_{eg}^{2}\left({\hat{\sigma }}^{\left(i\right)}+{\hat{\sigma }}^{(i)†}\right),$$

where $\mu_{mn}\equiv {\langle \hat{\mu }\rangle }_{mn}\left(R_{i}\right)$ and $\mu_{mn}^{2}\equiv {\langle {\hat{\mu }}^{2}\rangle }_{mn}\left(R_{i}\right)$ are the ***R***_***i***_ dependent dipole matrix elements and squared dipole moments between electronic states *m* and *n*, respectively. The total dipole moment operator $\tilde{\mu }$ after the CS transformation reads

(13) $$\tilde{\mu }=\sum_{i=1}^{N}{\hat{\mu }}^{\left(i\right)}-{\langle \hat{\mu }\rangle }_{0},$$

where ${\langle \hat{\mu }\rangle }_{0}$ is the ground state static dipole moment of the whole ensemble. The corresponding squared dipole operator is given by

(14) $${\tilde{\mu }}^{2}=\sum_{i=1}^{N}{\left({\hat{\mu }}^{\left(i\right)}\right)}^{2}-2{\hat{\mu }}^{\left(i\right)}{\langle \hat{\mu }\rangle }_{0}+\sum_{j\neq i}^{N}{\hat{\mu }}^{\left(i\right)}{\hat{\mu }}^{\left(j\right)}+{\langle \hat{\mu }\rangle }_{0}^{2}.$$

The first two terms are operators acting locally on each molecule. On the contrary, the third term of Eq. (14) describes an intermolecular interaction by directly connecting the dipole moment operators ${\hat{\mu }}^{\left(i\right)}$ and ${\hat{\mu }}^{\left(j\right)}$ of two molecules. This interaction of two molecules induced by DSE has been shown to play an important role in the description of molecular ensembles under VSC.^33,34^

The total wave function of the coupled ensemble is represented as a tensor product of the wave function of each molecule and the Fock states of the photon mode. Here, we truncate the wave function to a maximum of two excitations. Each molecule is, by definition, limited to a maximum of one excitation. The resulting product wave function for *N* molecules reads

(15) $$\begin{array}{l} |\text{Ψ};n_{p}\rangle :\{|G;0\rangle ,|G;1\rangle ,|E^{\left(i\right)};0\rangle ,\\ \;\;\;\;\;\;\;\;\;\;\;\;\;\;\;\;\ldots ,|G;2\rangle ,|E^{\left(i\right)};1\rangle ,\ldots ,|{ℰ}^{\left(i,j\right)};0,\ldots \}, \end{array}$$

where ∣*G*⟩ ≡ ∣*g*_1_, … ⟩ is the collective molecular ground state. The 2*N* states of the form ∣*E*^(*i*)^; *n*⟩ ≡ ∣*g*_1_, *g*_2_, *e*_*i*_, … ; *n*⟩ are described by a single excited molecule *i* and *n* photons, and *N(N* – 1) /2 additional states of the form ∣*ℰ* ^(*i*, *j*)^; 0⟩ ≡ ∣*g*_1_, *e*_*i*_, *e*_*j*_, … ; 0⟩ are characterized by two excited molecules.

In Eq. (16), we show the schematic structure of the matrix representing the light–matter interaction terms of *Ĥ*_*cs*_, which consist of linear dipole coupling and the DSE terms. Since the matrix is symmetric, only the upper triangle is shown, and prefactors are excluded for improved clarity. Since we are interested in the dynamics in the first excitation manifold, we do not show all coupling terms within the ∣*ℰ* ^(*i*, *j*)^; 0⟩ states,

(16) $$\begin{array}{l} \begin{array}{llllllllll} \;|G;0\rangle & |G;1\rangle & |E^{\left(1\right)};0\rangle & \;\cdots & |E^{\left(N\right)};0\rangle & |G;2\rangle & |E^{\left(1\right)};1\rangle & \;\cdots & |E^{\left(N\right)};1\rangle & |{ℰ}^{\left(i,j\right)};0\rangle \end{array}\\ \begin{array}{l} \langle G;0|\\ \langle G;1|\\ \langle E^{\left(1\right)};0|\\ \vdots \\ \langle E^{\left(N\right)};0|\\ \langle G;2|\\ \langle E^{\left(1\right)};1|\\ \vdots \\ \langle E^{\left(N\right)};1|\\ \langle {ℰ}^{\left(i,j\right)};0| \end{array}\left(\begin{array}{cccccccccc} {\left(\lambda \tilde{u}\right)}_{G}^{2} & 0 & 0 & \cdots & 0 & 0 & 0 & \cdots & 0 & 0\\ & {\left(\lambda \tilde{u}\right)}_{G}^{2} & \lambda {\tilde{u}}_{eg} & \cdots & \lambda {\tilde{u}}_{eg} & 0 & 0 & \cdots & 0 & 0\\ & & {\left(\lambda \tilde{u}\right)}_{E}^{2} & & {\left(\lambda \tilde{u}\right)}_{ee}^{2} & 0 & \lambda {\tilde{u}}_{ee} & & 0 & 0\\ & & & \ddots & & \vdots & & \ddots & & \vdots \\ & & & & {\left(\lambda \tilde{u}\right)}_{E}^{2} & 0 & 0 & & \lambda {\tilde{u}}_{ee} & 0\\ & & & & & {\left(\lambda \tilde{u}\right)}_{G}^{2} & \lambda {\tilde{u}}_{eg} & \cdots & \lambda {\tilde{u}}_{eg} & 0\\ & & & & & & {\left(\lambda \tilde{u}\right)}_{E}^{2} & & {\left(\lambda \tilde{u}\right)}_{ee}^{2} & \lambda {\tilde{u}}_{eg}\\ & & & & & & & \ddots & & \vdots \\ & & & & & & & & {\left(\lambda \tilde{u}\right)}_{E}^{2} & \lambda {\tilde{u}}_{eg}\\ & & & & & & & & & {\left(\lambda \tilde{u}\right)}_{ℰ}^{2} \end{array}\right) \end{array}.$$

To further reduce complexity, the rotating wave approximation (RWA) has been applied, which removes all rapidly oscillating terms.^52^ For validation, we performed benchmark calculations with and without the RWA; the results are shown in Sec. S1 of the supplementary material.

The linear dipole interactions create off-diagonal terms that can be categorized into two groups: the first group [highlighted in purple in Eq. (16)] corresponds to the conventional TC coupling terms that couple different electronic states. The second group of linear couplings [highlighted in green in Eq. (16)], which are not part of the standard TC Hamiltonian, couples different vibrational states within the same electronic state. This coupling term is zero for all states formed by the ensemble ground state due to the CS transformation. Note that vibrational states in the electronic ground state are coupled indirectly through the dependence of *â*_*cs*_ on ***R***. The DSE terms yield two different types of terms. The first type are the diagonal elements ${\left(\lambda \tilde{\mu }\right)}_{G}^{2},{\left(\lambda \tilde{\mu }\right)}_{E}^{2}$, and ${\left(\lambda \tilde{\mu }\right)}_{ℰ}^{2}$ that lead to a state-specific energy shift. The second group of DSE contributions (marked orange) connects states with the same photon number but with electronic excitations located on different molecules. These terms are a direct consequence of the intermolecular dipole–dipole interaction in Eq. (14). The corresponding matrix elements have the following form:

(17) $${\left(\lambda \tilde{\mu }\right)}_{ee}^{2}=\lambda^{2}\mu_{eg}^{\left(i\right)}\left(R_{i}\right)\mu_{ge}^{\left(j\right)}\left(R_{j}\right),$$

and show that the molecular excitations can be exchanged through the cavity mode by means of the DSE. A detailed derivation of all interaction terms for the case of *N* molecules can be found in Sec. S1 of the supplementary material. Note that increasing the number of molecules in this model increases the size of the matrix *Ĥ*_*cs*_ but does not introduce new types of interaction.

**Fig. 2:**
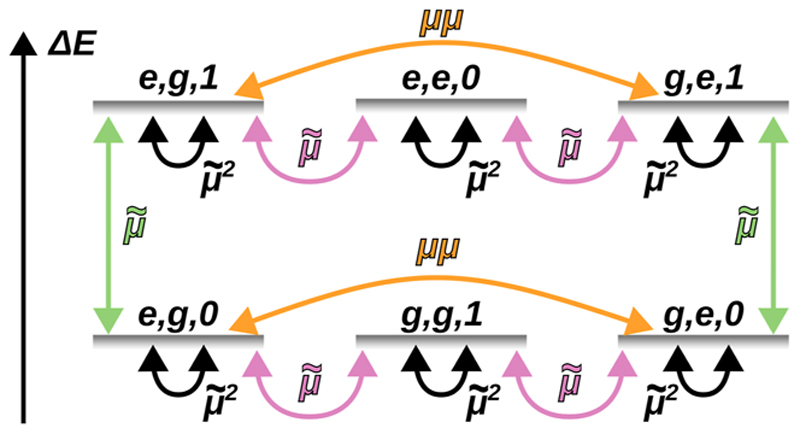
Scheme for the couplings between the bare-states of a coupled system of two molecules and a cavity mode, where $\tilde{\mu }$ stands for the static dipole coupling, ${\tilde{\mu }}^{2}$ stands for the DSE shifts, and *μμ* stands for the intermolecular excited states couplings arising from the DSE.

All relevant interactions are depicted schematically for the single excitation manifold in Fig. 2. All states within the first excitation manifold are directly coupled by either the linear dipole interaction (pink) or the intermolecular DSE contribution (orange).

## III Computational Details

All electronic structure calculations of MgH^+^ are performed with the MOLPRO program package^53^ version 2021.2^54–57^ at the CAS(6/9)/MRCI/aug-cc-pVQZ^58^ level of theory with six active electrons in nine orbitals.^59–61^ In total, five electronic states are included in the state-average procedure. The static and transition dipole moments are obtained directly from MOLPRO, while the squared dipole moments are calculated using a resolution of identity approach^62–64^

(18) $$\mu_{ij}^{2}(R)\approx \sum_{k=1}^{5}\mu_{ik}(R)\mu_{kj}(R).$$

Here *i, j*, and *k* refer to the electronic states, and the sum runs over all five states involved in the state averaging procedure (see Sec. S5 of the supplementary material for details on the convergence of the squared dipole moments).

**Fig. 3:**
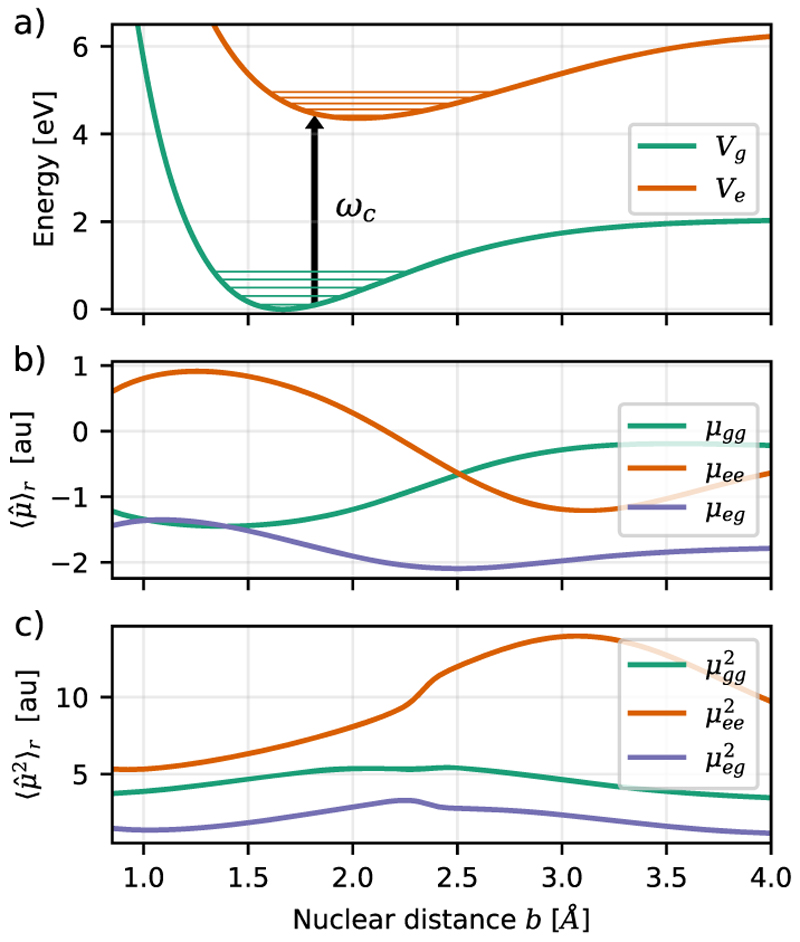
(a) Bare ground (Σ_0_ ≡ *g*) and excited (Σ_1_ ≡ *e*) electronic potential energy surfaces of MgH^+^. (b) Static and transition dipole moment functions (⟨*μ*⟩_*gg*_, ⟨*μ*⟩_*ee*_, and ⟨*μ*⟩_*ge*_) along the molecular bond (*z* axis) and (c) corresponding squared dipole moment functions (⟨*μ*^2^⟩_*gg*_, ⟨*μ*^2^⟩_*ee*_, and ⟨*μ*^2^⟩_*eg*_).

All necessary properties (see Fig. 3), such as PESs and dipole moments, are calculated on a coarse grid between *R* = 0.8 Å and *R* = 4.0 Å and interpolated to a finer grid. Both states exhibit a static dipole moment and a transition dipole moment along the molecular bond; see Fig. 3(b). The corresponding squared dipole moments calculated using the resolution of the identity approach are shown in Fig. 3(c). The two- and three-dimensional surfaces for the ensemble of two and three MgH^+^ molecules are constructed from the molecular PESs. Details of all three grids can be found in Table I.

The cavity frequency *ω*_*c*_ = 4.322 eV is chosen to be resonant to the energy difference between the first vibrational states of each potential (∣*g, v* = 0⟩ → ∣*e, v* = 0⟩ transition), which is indicated by the arrow in Fig. 3(a). In addition to the standard TC coupling schema and our ETC Hamiltonian, we extend the molecular TC model only with static dipole moments or DSE contributions. The latter two are only used for benchmarking purposes.

To evaluate the influence of the new terms introduced in the molecular ETC model, we compare the dynamics of small molecular ensembles to the molecular TC model. For all Hamiltonians, the excited state dynamics has been simulated by numerically propagating the time-dependent Schrödinger equation with the Arnoldi propagation scheme.^65^ The vibrational eigenfunctions of the uncoupled potentials are obtained using the imaginary time propagation method.^66^ The optimized ground state wave function ∣*G*, 0⟩ is used to initiate coupled dynamics in the ∣*G*, 1⟩ state. The grid-based quantum dynamics simulations are performed with the QDng quantum dynamics program package.^67^ All calculations were performed in a reproducible environment using the Nix package manager together with NixOS-QChem^68^ (commit f803c22214) and Nixpkgs (nixpkgs, 23.05, commit 5550a85a08).

## IV Population Dynamics Of Different Light–Matter Hamiltonians

**Fig. 4:**
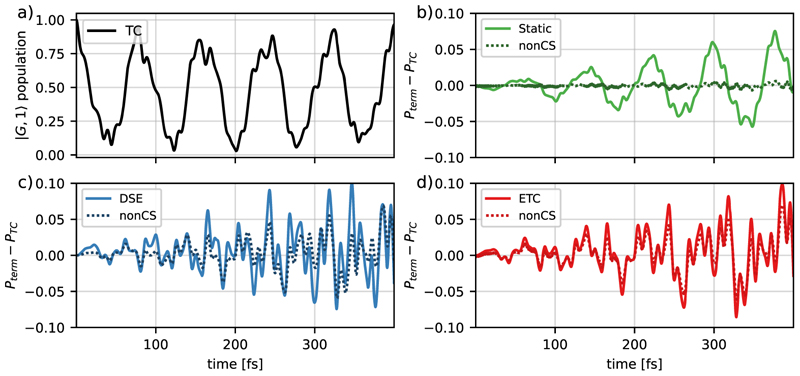
Time evolution of the ∣*G*; 1⟩ population for a single MgH^+^ molecule coupled to the cavity mode for (a) the molecular TC model, (b) population differences to the molecular TC model by the inclusion of only the static dipole coupling interactions terms, (c) only the DSE terms, and (d) the molecular ETC model considering both terms. The corresponding results without the CS transformation are indicated by dotted lines. All propagations were performed with a cavity resonance frequency of *ω*_*c*_ = 4.322 eV and a coupling strength of *λ*_*c*_ = 6.9 × 10^−3^ a.u.

Let us first analyze the effect of the additional terms in the molecular ETC model on the dynamics of a single MgH^+^ molecule and compare its results with those of the standard molecular TC model. These terms are the static dipole moment coupling and the contribution of DSE, as well as the changes induced by the CS transformation. Figure 4 shows the population dynamics of ∣*G*; 1⟩ using the standard molecular TC model, as well as the changes in the population caused by adding the additional terms mentioned above to the light–matter Hamiltonian and the differences caused by the CS transformation.

For the molecular TC model, the population dynamics of the ∣*G*; 1⟩ state is characterized by Rabi oscillations with a period of 80 fs, which corresponds to an effective Rabi frequency of 52 meV/*ħ* [see Fig. 4(a)]. The fine structure of the oscillations is caused by the motion of the nuclear wave packet in the ∣*G*; 1⟩ and the ∣*E*; 0⟩ states. By including additional coupling terms in the Hamiltonian, the Rabi oscillations are preserved, but the resulting population dynamics are changed compared to the molecular ETC simulation, which can be seen in Figs. 4(b) and 4(c). The observed differences are mainly caused by a difference in the observed Rabi frequency, which also explains the increase in the difference with increasing simulation time. Taking into account only the static dipole interaction terms [Fig. 4(b)] or only the DSE contributions [Fig. 4(c)], the frequency difference leads to a maximum population difference of 0.075 and 0.10, respectively, within the initial 400 fs. The population difference, when only the static dipole interaction terms are included, shows a slow oscillating pattern with a frequency comparable to the Rabi frequency, whereas for the case of only DSE contributions much faster oscillations are observed. For the molecular ETC model [Fig. 4(d)] these two contributions are combined resulting in a maximum population difference of 0.10 in the same time window. Figures 4(b) through 4(d) also show the cases where the CS transformation is not applied. In the case of the molecular ETC Hamiltonian [Fig. 4(d)], dropping the CS transformation results in a negligible error. However, if the molecular ETC Hamiltonian is only extended by static dipole terms the result is almost identical to the molecular TC model [see Fig. 4(b)]. In contrast, comparing the DSE-only results [Fig. 4(c)] with and without the CS transformation gives a comparable result. This behavior, and the negligible error for the molecular ETC Hamiltonian, indicates that the population difference between the ETC and TC models is mostly determined by the DSE contribution.

**Fig. 5:**
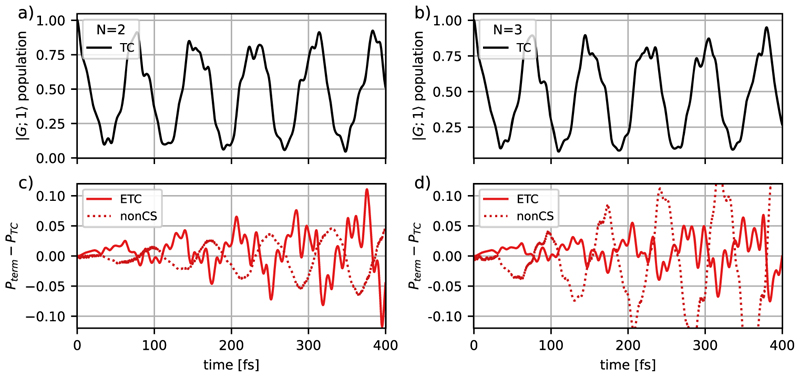
Time evolution of the ∣*G*; 1⟩ population for two MgH^+^ molecules, (a) and (c), and for three MgH^+^ molecules, (b) and (d), coupled to the cavity mode. Figures (a) and (b) show the results for molecular TC. Figures (c) and (d) show the difference in the ∣*G*; 1⟩ population between the molecular TC and ETC Hamiltonian, which includes the static dipole coupling interactions and the DSE terms. The corresponding results without the CS transformation are shown by dotted lines. All simulations were performed with a cavity excitation of *ω*_*c*_ = 4.322 eV and a collective coupling strength of *λ*_*c*_ = 6.9 × 10^−3^ a.u.

The population dynamics of the ∣*G*; 1⟩ state is visualized in Fig. 5 for the case of two and three MgH^+^ molecules coupled to a cavity to examine how the molecular ETC Hamiltonian and the CS transformation change the results of the standard molecular TC model. The molecules are assumed to be oriented in parallel. The population dynamics obtained with the molecular ETC model and the observed Rabi oscillations are qualitatively similar for one, two, and three MgH^+^ molecules. Note that the collective coupling strength is kept constant due to the rescaling of the single-particle coupling strength by $1/\sqrt{N_{Mol}}$ [see Eq. (3)]. However, the differences between molecular TC and the ETC Hamiltonian are affected by the increase in the number of molecules. Going from a single molecule [Fig. 4(c) solid line] to two molecules [Fig. 5(c) solid line], the deviation between the TC model and the ETC model remains comparable, while for three molecules [Fig. 5(d) solid line] the size of the deviation is reduced. The corresponding population differences due to the inclusion of only the DSE or only the static dipole moment are shown in Fig. S3 in the supplementary material. Interestingly, we observe that the influence of the static dipole moment contribution decreases with the number of molecules faster than the DSE, which can be attributed to the CS transformation. If the transformation is not performed [see Figs. 5(c) and 5(d), dotted lines] the difference between the molecular TC and ETC model becomes larger for an increasing number of molecules, which can be explained by the increasing total dipole moment of the ensemble. In summary, in the case of a few molecules, the non-trivial interplay of DSE contributions and the presence of static dipole moments define a situation where none of the terms can be simply neglected. In particular, intermolecular dipole–dipole interactions due to DSE in the molecular ETC Hamiltonian [Eq. (17)] play an important role; see Sec. S1 in the supplementary material. The error with respect to the CS transformation [see Figs. 5(c) and 5(d), dotted lines] also becomes larger for an increasing number of molecules, which can be explained by the increasing total dipole moment of the ensemble.

## V Polaritonic Absorption Spectra

**Fig. 6:**
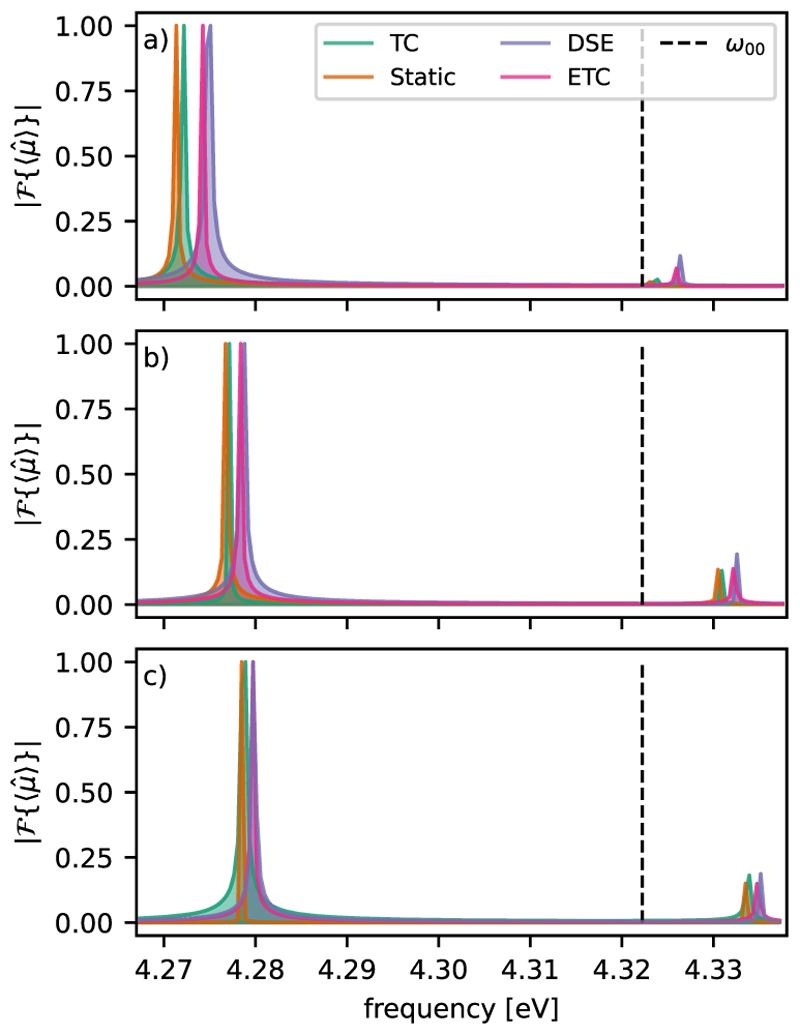
Relevant part of the polaritonic absorption spectra for (a) 1, (b) 2, and (c) 3 MgH^+^ molecules coupled to a cavity for different Hamiltonians. The spectra show the LP and UP transitions and are calculated by the Fourier transformation of the expectation value of the total dipole moment. The different models are the molecular ETC model (pink), the molecular TC model with only static dipole moments (blue), the molecular TC model with only DSE contribution, and the molecular TC model (green). The dashed line corresponds to the first vibrational resonance between the electronic states. All simulations were performed with a cavity excitation of *ω*_*c*_ = *ω*_00_ = 4.322 eV and a coupling strength of *λ*_*c*_ = 6.9 × 10^−3^ a.u.

We next compare the corresponding absorption spectra for the four different molecular Hamiltonians of the coupled molecular–cavity system. In Fig. 6, the spectra of the LP and UP transitions are shown for the cases of one, two, and three MgH^+^ molecules coupled to a cavity resonant with the ∣*g, v* = 0〉 → ∣*e, v* = 0⟩ transition. The complete absorption spectra and detailed analysis of all features are provided in Sec. S2 of the supplementary material. To obtain the spectra of the coupled molecular–cavity system, a superposition $(|G,0\rangle +|G,1\rangle )/\sqrt{2}$ was propagated for 10 ps, and the expectation value of the total dipole moment was Fourier transformed.

The observed Rabi splitting of about ≈53 meV is almost the same for the four light–matter Hamiltonians, as well as for *N* = 1, 2, 3. Regardless of the number of molecules resonantly coupled to the cavity, the LP and UP transitions are strongly asymmetric and redshifted with respect to *ω_c_* and the bare molecular ∣*g, v* = 0⟩ → ∣*e, v* = 0⟩ transition. Interestingly, this asymmetry is already present when using the standard molecular TC model Hamiltonian (Fig. 6, green). Including the static dipole moment leads to an increased redshift (Fig. 6, orange), while including only the DSE (Fig. 6, blue) leads to a decreased redshift of the LP and UP signals. Consistent with the results for population dynamics, the light–matter Hamiltonian (Fig. 6, pink) is closer to the molecular TC model. However, the observed differences between the different light–matter Hamiltonians are getting smaller as more molecules are included [see Figs. 6(a)–6(c)].

From Fig. 6, we can conclude that the observed redshift and asymmetry in the spectrum are not caused by the DSE or the influence of the static dipole moments, since they are already present in the molecular TC model. Note that similar redshifts have also been observed in electronic structure calculations, where the effect of the electric field mode has been incorporated.^36^ In these cases, the redshift is a consequence of matter polarization. In the following, we demonstrate that the observed redshift in the molecular TC and ETC models is caused by the molecular Franck–Condon (FC) factors. To quantify the asymmetry, we calculate the eigenvalues of the coupled system in dependence on the relative shift in the nuclear coordinate *R* of the PESs for *V*_*g*_ and *V*_*e*_. The relative shift between the minima of *V*_*g*_ and *V*_*e*_ is defined as *δb*. The resulting modified molecular Hamiltonian is coupled to the cavity mode that is resonant with the ∣*g, v* = 0⟩→ ∣*e, v* =0⟩ transition to yield the molecular TC Hamiltonian together with the transition dipole moment of MgH^+^. By diagonalizing the resulting Hamiltonian, we obtain the polariton states for the shifted potential set-up as a function of *δb*.

**Fig. 7:**
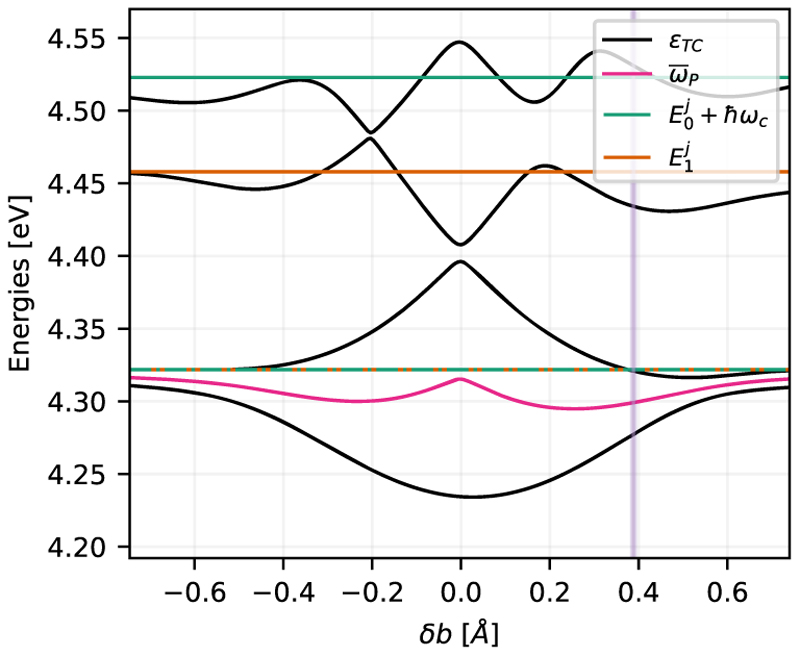
Polaritonic eigenstates (*ϵ*_*TC*_) for the coupled system formed by the vibrational states of the single molecule electronic potentials *V*_*g*_ and *V*_*e*_, as a function of the relative position *δb* between the two minima, for a coupling strength of *λ*_*c*_ = 6.9 × 10^−3^ a.u. and a cavity frequencies of 4.322 eV. The green and orange lines indicate the uncoupled vibrational eigenstates *E*_*i*_, and the pink line indicates the average frequency ${\bar{\omega }}_{P}$ of the LP and UP eigenstates. The purple vertical line indicates the natural distance between the potentials (0.388 Å).

The first four resulting polariton states are shown in Fig. 7 (black lines). The asymmetry of the eigenvalues around the field free transition is quantified by the average energy ${\bar{\omega }}_{P}$ of the LP and UP states (pink lines). The uncoupled energies are shown for reference as green and orange lines in Fig. 7. The magnitude and asymmetry of the Rabi splitting strongly depend on the relative shift between the coupled potentials. Even if the potential minima are aligned (*δb* = 0 Å), LP and UP are not perfectly symmetric. Due to the different vibrational frequencies and anharmonicity parameters of the potentials *V*_*g*_ and *V*_*e*_, the FC matrix is not fully diagonal for *δb* = 0 Å. For larger values of ∣*δb*∣, the overlap of the wave function vanishes and effectively decreases the transition dipole moment, resulting in a smaller Rabi splitting. As described in Sec. S3 of the supplementary material, this asymmetry in the Rabi splitting induced by the FC factors can even be observed in the case of two identical harmonic potentials. Higher lying vibrational states begin to mix into polariton states and, thus, lead to a shift in the eigenvalues. Thus, the case of perfectly symmetric Rabi splitting in molecular ESC seems to be an exception rather than the standard case, since anharmonicity and shifted potential energy surfaces are common in molecular systems.

## VI Effective Molecular Tavis–Cummings Model

The main challenge of the molecular TC model is the exponential scaling of the wave function with respect to the number of molecules. Simulating the full dynamics, including all vibrational degrees of freedom, becomes prohibitively expensive in terms of computational effort. Simplifying the description of the matter and replacing the molecules with effective TLSs can greatly reduce the computational cost.

In the following, we derive an effective model based on an ensemble of TLSs coupled to a single cavity mode, starting from the molecular ETC Hamiltonian after the CS transformation. The effect of the static dipole moments and the DSE is thus preserved. Each molecule in the ensemble is replaced by a two-level emitter defined by the two electronic states. The matter Hamiltonian shown in Eq. (10) is simplified to $H_{M}=\omega_{\text{eg}}{\hat{\sigma }}^{†}\hat{\sigma }$, where *ω*_*eg*_ is the energy difference between the ∣*g, v* = 0⟩ → ∣*e, v* = 0⟩ transition. The nuclear position-dependent dipole moment and dipole moment squared operators $\tilde{\mu }$ and ${\tilde{\mu }}^{2}$ of each molecule are replaced by the corresponding expectation values $\langle \tilde{\mu }\rangle$ and $\langle {\tilde{\mu }}^{2}\rangle$ at the FC point for each of the electronic states. A detailed derivation of the general TLS model Hamiltonian and all of its coupling terms can be found in Sec. S4 of the supplementary material.

**Fig. 8:**
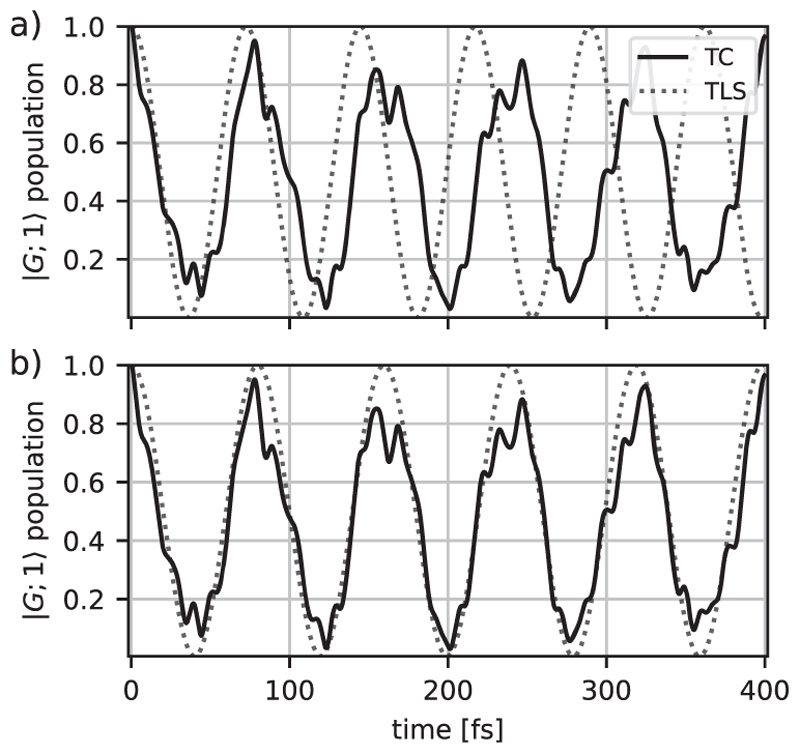
Comparison of propagation of a single molecule system to a single TLS using the TC model (a) with the same cavity parameters: a coupling strength of *λ*_*c*_ = 6.9 × 10^−3^ a.u. and a cavity resonant to the TLS frequency excitation of $\omega_{eg}^{TLS}=4.322\;\text{eV}$, and (b) with optimized TLS parameters: a coupling strength of *λ*_*c*_ = 6.25 × 10^−3^ a.u., a level resonance of $\omega_{eg}^{TLS}=4.297\text{eV}$, and a cavity detuning of −3.59 meV.

To evaluate the validity of the TLS approximation, we compare the dynamics of the ∣*G*; 1⟩ population in the case of one MgH^+^ molecule coupled to a cavity with the results obtained for a TLS using the TC model for both. The results are shown in Fig. 8. If the resonant frequency of the cavity and the energy difference of the levels are not modified $\left(\omega_{c}=\omega_{eg}^{TLS}=4.322\;\text{eV}\right)$, the temporal evolution of the ∣*G*; 1⟩ state population is different [see Fig. 8(a)]. The two notable differences are an increase in Rabi frequency for the TLS approximation and the absence of fine structure in the oscillations caused by the vibrational motion. The underlying reason for this discrepancy can be found in the construction of the TLS model: the absence of vibrational degrees of freedom and a non-diagonal FC matrix results in a symmetric Rabi splitting (see Sec. V). Thus, the energetics, as well as the population dynamics, are different in the TLS model and the molecular system when the same cavity parameters are used. To improve the TLS model, we optimize its parameters to mimic the energetics of the molecular polaritonic states. These optimized parameters can be obtained by fitting the TLS polariton energies to the molecular absorption spectra. A detailed explanation of this optimization process can be found in Sec. S4 of the supplementary material. The population dynamics of the ∣*G*; 1⟩ state using the optimized TLS parameter ($\omega_{eg}^{TLS}=4.297$ *eV* and a cavity detuning of −3.59 meV) compared to the molecular simulation is shown in Fig. 8(b). With the optimized parameters, the TLS model qualitatively reproduces the population dynamics of the molecular system and exhibits an identical Rabi frequency.

**Fig. 9:**
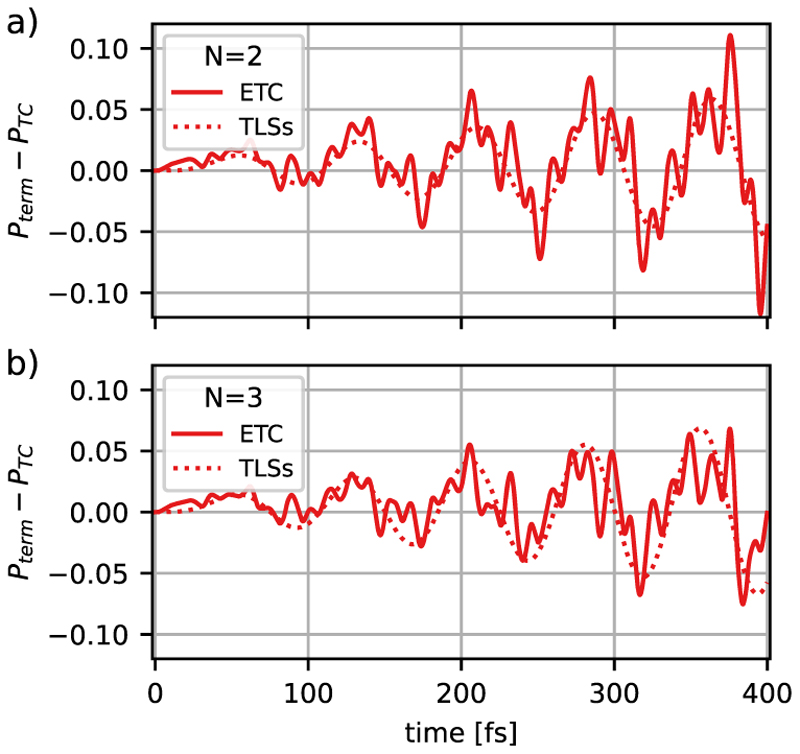
State ∣*G*; 1⟩ population dynamics of the optimized TLSs model and the molecular simulations for (a) two and (b) three MgH^+^ molecules coupled to the cavity mode, comparing the full-TLSs and ETC model Hamiltonian population differences to the corresponding TLSs or molecular TC model.

To extend the validity of the TLS approximation, we compare the population difference between the TC and the extended models in the molecular and optimized TLS cases. The results for two and three MgH^+^ molecules/emitters are shown in Fig. 9. The qualitative agreement of the TLS improves as the number of molecules increases. Similarly to Sec. IV, the influence of nuclear wave packet dynamics becomes smaller with an increasing number of molecules since the photonic excitation is evenly distributed over more molecules, resulting in less vibrational excitation per molecule. It should be noted that the optimized parameters of the TLS models are slightly different for one, two, and three MgH^+^ molecules (see Table S1 in the supplementary material). However, we observe a tendency toward values close to the original molecular parameters as the number of molecules increases.

**Fig. 10:**
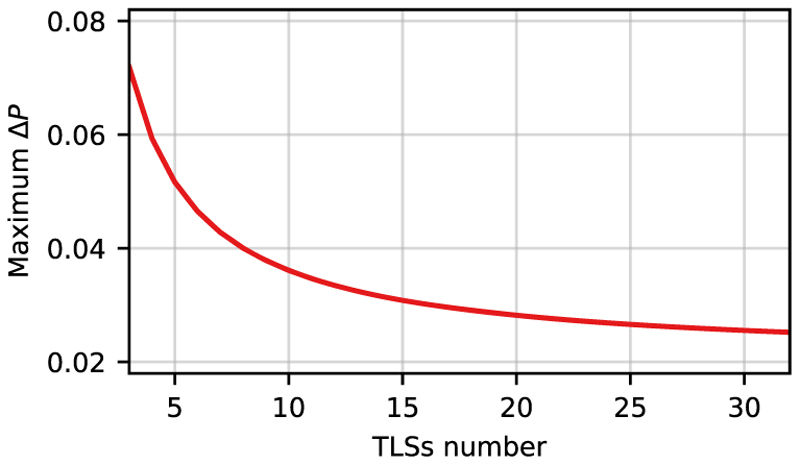
Maximum ∣*G*; 1⟩ population difference between the TLS-ETC model Hamiltonian and the TC model Hamiltonian after 400 fs as a function of the number of TLSs in the ensemble.

Based on these results, we assume that our TLS model with the optimized parameters for 3 MgH^+^ molecules is capable of capturing the essential features of the coupled system dynamics for the general *N* molecule situation. The TLS model allows us to simulate larger systems, and we chose a maximum of 33 emitters to demonstrate convergence. In Fig. 10, the maximum deviation of the population difference between the TLS-ETC and TC model Hamiltonians within 400 fs is plotted as a function of the TLS number *N*. This maximum deviation is given by

(19) $$\text{max}\;\text{Δ}P(t,N)=\text{max}\left(\left|P_{\text{ETC}}-P_{\text{TC}}\right|\right),$$

where *P*_ETC_ and *P*_TC_ are the state ∣*G*; 1⟩ populations for the different Hamiltonian propagations. Their maximum difference is calculated by fitting the local maxima to linear regression and deriving its value at time *t*. The maximum deviation in the population decreases with the increasing number of TLSs coupled to the cavity mode, as shown in Fig. 10. An increase in *N* leads to an increase in the energy shifts of the states and couplings between excited states in Eq. (16). However, due to rescaling of the coupling strength, this increase is fully compensated, and, as a consequence, the difference between the TLS-ETC and TC model Hamiltonians is inversely proportional to *N*. To confirm this behavior, a function of max Δ*P* = 0.020 + 0.151*N*^−0.970^ has been fitted to the calculated curve in Fig. 10. This fit confirms that the maximum difference decays with an exponent of −0.970 and converges to a finite difference of 0.020 for large *N*.

**Fig. 11:**
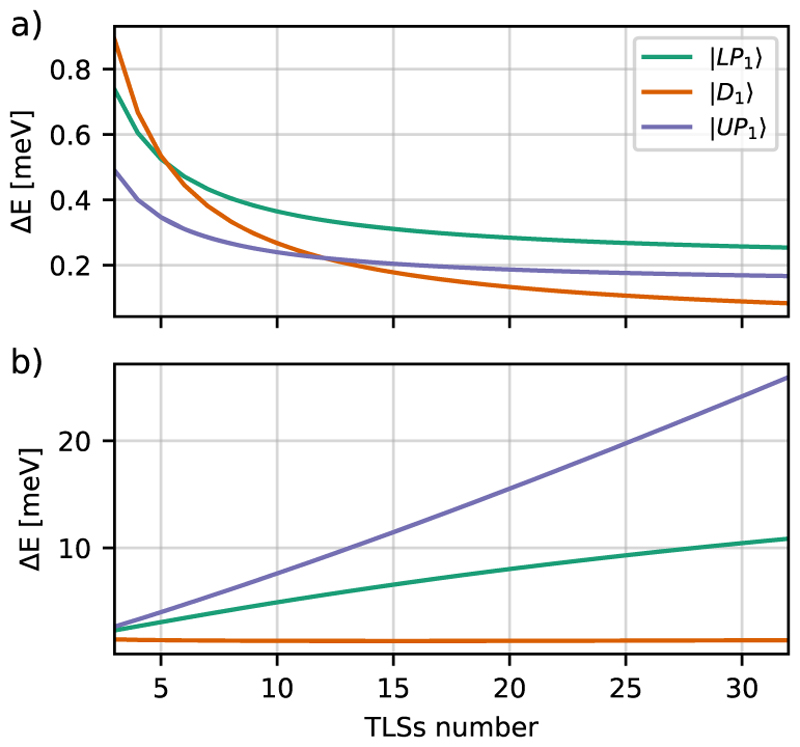
TLS eigenvalue differences between diagonalized TLS-ETC and TC models as functions of the system size for (a) the CS transformed and (b) non CS transformed systems. A scaled coupling strength of *λ*_*c*_ = 6.52 × 10^−3^ a.u., a level frequency difference of *ω*_*eg*_ = 4.312 eV, and a cavity detuning by 11.6 meV. *UP*_1_ stands for the upper polariton of the first excitation manifold, *LP*_1_ stands for the lower polariton, and *D*_1_ stands for the dark states.

In addition to studying population dynamics, we examine the eigenenergies of the TLS-ETC model Hamiltonian to determine the influence of the CS transformation on the polaritonic states, as well as the effect of including static dipole moments and DSE terms. By diagonalizing the *N*-TLSs Hamiltonian, we obtain two polariton states, ∣*LP*⟩, ∣*UP*⟩, and *N* − 1 dark states summarized as ∣*D*_1_⟩. The variation in the eigenenergies of the TLS-ETC and TC model Hamiltonians is given by

(20) $$\text{Δ}E_{S}=\langle {\hat{H}}_{\text{ETC}}^{\text{TLS}}\rangle S-\langle {\hat{H}}_{\text{TC}}^{\text{TLS}}\rangle S,$$

where *S* is a particular polariton eigenstate of the respective Hamiltonian. In Fig. 11(a), these energy differences between the eigenenergies of the TLS-ETC and TC model Hamiltonians are shown as a function of the TLS number. All three curves, the upper and lower polarition states, as well as the dark states, have also been fitted to the following functions:

$$\begin{array}{l} \text{Δ}E_{\text{LP}}=0.203\text{meV}+1.595\text{meV}\;N^{-0.995},\\ \text{Δ}E_{\text{D}}=0.000\text{meV}+2.672\text{meV}\;N^{-1.000},\\ \text{Δ}E_{\text{UP}}=0.134\text{meV}+1.077\text{meV}\;N^{-1.008}. \end{array}$$

The energy differences of all states are approximately inversely proportional to the number of TLSs, similar to the trend observed in Fig. 10. This is consistent with a *λ*^2^ square scaling, as it appears in the DSE term. The difference between the TLS-ETC and TC model Hamiltonians converges to zero for the dark states. However, for the LP state and the UP state, it converges for large *N* to finite values of 0.203 and 0.134 meV, respectively. This trend is consistent with the redshift and asymmetry observed in the spectra in Fig. 6. The covariance matrices for the fitted curves of maximum Δ*P* and Δ*E*_*S*_ are shown in Sec. S4 of the supplementary material.

Figure 11(b) shows the energy difference for TLS without the CS transformation. The energies of the two bright polaritonic states diverge with an increase in *N*. Note that, similar to Figs. 5(b) and 5(d), the TLS represent molecules that are aligned in parallel. This results in an increasing total dipole moment with an increasing *N* and causes divergent behavior. The dark state energies are not affected by the CS transformation, as they are decoupled from the cavity mode and have no photonic contribution.

## VII Conclusion

We have extended the molecular TC model to include both state-specific static dipole moments and the DSE contribution in the light–matter Hamiltonian. Starting from the non-relativistic Pauli–Fierz Hamiltonian, we derived a light–matter Hamiltonian in the CS basis and used the RWA to describe molecules under ESC. The studied molecular system consists of a varying number of MgH^+^ molecules coupled to a single-photon mode of an optical cavity resonant with the first electronic transition in MgH^+^. By analyzing the difference in the population dynamics obtained with the molecular TC model and the generalized molecular ETC, we could identify changes independent of the number of molecules. The deviations caused by either the static dipole moments or the DSE contributions are significantly different and give rise to the overall difference in the molecular ETC model. Thus, both components are essential to describe molecules coupled to a cavity. In line with the literature,^31–37^ we can, therefore, emphasize that the DSE should not be neglected, even in the ESC regime. Although the discrepancy between the molecular TC and ETC decreases for larger *N*, we could show that it converges to finite values. Another aspect that has been discussed in the literature, mainly for *ab initio* methods,^39,43,46^ is the use of the CS transformation for systems with a static dipole moment. We could show that the CS transformation becomes relevant when the total dipole moment of the molecular ensemble is nonzero.

The DSE contribution itself does not depend on the number of photons and, therefore, should be affected only indirectly by the photon loss of the cavity. However, photon decay and dissipation can affect the dynamics of molecular systems under ESC quite drastically.^59,69^ Thus, it would be an interesting next step to study the interplay of DSE and photon loss.

Analysis of the polaritonic absorption spectra of coupled MgH^+^-cavity systems revealed that LP and UP are strongly red-shifted and are asymmetric in intensity. This phenomenon is independent of the exact model used and could even be observed for harmonic potentials. We could identify this shift as a result of the molecular FC factors, which depended on the relative change in the equilibrium bond length between the ground and excited states. In the VSC regime, such a redshift is associated with self-consistent treatment of the electronic structure problem,^33,36,37^ which is not the case in our ESC simulation.

Furthermore, we investigated the possibility of representing the molecules as TLSs with only two electronic states each and without nuclear degrees of freedom. We constructed an ensemble of TLSs to estimate the population dynamics of larger ensembles of up to 33 MgH^+^ molecules based on the presented molecular ETC model. Such a model could be used to describe larger ensembles where the nuclear motion is not important and does not lead to reactions or nuclear rearrangements.

## Supplementary Material

Supplementary Material

## Figures and Tables

**Table I:** Details of the grid and simulation parameters. The number of points *N* is given for each dimension of the grid. The minimum and maximum values of the internuclear distance *R*, the propagation time, and the time step are given in atomic units.

	*N*	*R*_min_ (a.u.)	*R*_max_ (a.u.)	Δ*t* (a.u.)	*t*_max_(fs)
(MgH^+^)_1_	128	1.61	7.56	5.0	500
(MgH^+^)_2_	64 × 64	1.61	6.61	5.0	500
(MgH^+^)_3_	64 × 64 × 64	1.61	6.61	5.0	500

## Data Availability

The data that support the findings of this study are available from the corresponding author upon reasonable request.
