# Supplementary material for: Extending the Tavis–Cummings model for molecular ensembles—Exploring the effects of dipole self-energies and static dipole moments
Source: J Chem Phys. Author manuscript; Available in PMC 2024 Aug 9. (PMC7616353; doi:10.1063/5.0214362)
Supplement: Supplementary Material [file EMS197951-supplement-Supplementary_Material.pdf]

Supporting Information: Extending the Tavis-Cummings model for molecular ensembles – Exploring the effects of dipole self energies and static dipole moments

Lucas Borges, Thomas Schnappinger,* and Markus Kowalewski†

Department of Physics, Stockholm University,

AlbaNova University Center, SE-106 91 Stockholm, Sweden

* thomas.schnappinger@fysik.su.se

† markus.kowalewski@fysik.su.se

CONTENTS

S1. The generalized molecular Tavis-Cummings model 3

S2. Absorption Spectra 9

S3. Polaritonic Franck-Condon Factors 11

S4. Derivation of the effective molecular Tavis-Cummings model 15

S5. Resolution of identity approach for the squared dipole operators 20

References 20

S1. THE GENERALIZED MOLECULAR TAVIS-CUMMINGS MODEL

After the length gauge and the coherent state (CS) transformation, the interaction part of the Pauli-Fierz Hamiltonian is given by:

$$\hat{H}_{int} = -\sqrt{\frac{\omega_c}{2}}(\hat{a}^\dagger + \hat{a})\,(\lambda\tilde{\mu}) + \frac{1}{2}\,(\lambda\tilde{\mu})^2\,, \tag{S1}$$

The total dipole moment operator $\tilde{\mu}$ of a ensemble of N molecules after the CS transformation reads:

$$\tilde{\mu} = \sum_{i=1}^{N} \hat{\mu}^{(i)} - \langle\hat{\mu}\rangle_0\,, \tag{S2}$$

where $\langle\hat{\mu}\rangle_0$ is the ground state permanent dipole moment of the whole ensemble and $\hat{\mu}^{(i)}$ is the dipole moment operator of the individual molecule in the nuclear subspace:

$$\hat{\mu}^{(i)} = \mu_{gg}\hat{\sigma}^{(i)}\hat{\sigma}^{(i)\dagger} + \mu_{ee}\hat{\sigma}^{(i)\dagger}\hat{\sigma}^{(i)} + \mu_{eg}\left(\hat{\sigma}^{(i)} + \hat{\sigma}^{(i)\dagger}\right)\,, \tag{S3}$$

where $\mu_{mn} \equiv \langle\mu\rangle_{mn}(\boldsymbol{R_i})$ are the $\boldsymbol{R_i}$ dependent dipole matrix elements between electronic states m and n respectively. The corresponding total squared dipole operator is given by

$$\tilde{\mu}^2 = \sum_{i=1}^{N}\left(\hat{\mu}^{(i)}\right)^2 + \hat{\mu}^{(i)}\left(\sum_{j\neq i}^{N}\hat{\mu}^{(j)} - 2\,\langle\hat{\mu}\rangle_0\right) + \langle\hat{\mu}\rangle_0^2\,. \tag{S4}$$

Here $\left(\hat{\mu}^{(i)}\right)^2$ is the squared dipole operator of the individual molecule in the nuclear subspace:

$$\left(\hat{\mu}^{(i)}\right)^2 = \mu_{gg}^2\hat{\sigma}^{(i)}\hat{\sigma}^{(i)\dagger} + \mu_{ee}^2\hat{\sigma}^{(i)\dagger}\hat{\sigma}^{(i)} + \mu_{eg}^2\left(\hat{\sigma}^{(i)} + \hat{\sigma}^{(i)\dagger}\right)\,, \tag{S5}$$

where $\mu_{mn}^2 \equiv \langle\mu^2\rangle_{mn}(\boldsymbol{R_i})$ are the squared dipole moments dependent on $\boldsymbol{R_i}$ between electronic states m and n respectively.

In the following derivation of the coupling terms in the generalized molecular Tavis-Cummings model we take advantage of the fact that the individual molecular wave functions $\left|g^{(i)}\right\rangle$ and $\left|e^{(i)}\right\rangle$ as well as the bare Fock states are orthonormal and the individual molecules are non-interacting. For brevity, prefactors are omitted, and the same color schema as in the manuscript is used to highlight relevant contributions. In the bare-state basis truncated to a maximum of two excitations, the first term in Eq. (S1) leads to linear dipole coupling terms between states in which the photonic excitation, i.e. the Fock state, changes. The

first type of linear coupling term connects different molecular ensemble states:

$$\langle G;0|\,(\hat{a}^\dagger+\hat{a})\tilde{\mu}\,\big|E^{(a)};1\big\rangle = \big\langle g^{(a)}\big|\,\hat{\mu}^{(a)}\,\big|e^{(a)}\big\rangle + \big\langle g^{(a)}\big|e^{(a)}\big\rangle(\dots) = \mu_{eg}^{(a)} \tag{S6}$$

$$\langle G;1|\,(\hat{a}^\dagger+\hat{a})\tilde{\mu}\,\big|E^{(a)};0\big\rangle = \big\langle g^{(a)}\big|\,\hat{\mu}^{(a)}\,\big|e^{(a)}\big\rangle + \big\langle g^{(a)}\big|e^{(a)}\big\rangle(\dots) = \mu_{eg}^{(a)} \tag{S7}$$

$$\langle G;2|\,(\hat{a}^\dagger+\hat{a})\tilde{\mu}\,\big|E^{(a)};1\big\rangle = \big\langle g^{(a)}\big|\,\hat{\mu}^{(a)}\,\big|e^{(a)}\big\rangle + \big\langle g^{(a)}\big|e^{(a)}\big\rangle(\dots) = \mu_{eg}^{(a)} \tag{S8}$$

$$\big\langle E^{(a)};1\big|\,(\hat{a}^\dagger+\hat{a})\tilde{\mu}\,\big|\mathcal{E}^{(a,b)};0\big\rangle = \big\langle g^{(b)}\big|\,\hat{\mu}^{(b)}\,\big|e^{(b)}\big\rangle + \big\langle g^{(b)}\big|e^{(b)}\big\rangle(\dots) = \mu_{eg}^{(b)} \tag{S9}$$

The second type of liner dipole interactions couple different vibrational states within the same electronic state:

$$\big\langle E^{(a)};1\big|\,(\hat{a}^\dagger+\hat{a})\tilde{\mu}\,\big|E^{(a)};0\big\rangle = \mu_{ee}^{(a)} + \sum_{b\neq a}^{N}\mu_{gg}^{(b)} - \langle\hat{\mu}\rangle_0 \tag{S10}$$

This type of coupling term is zero for all states formed by the ensemble ground state due to the CS transformation. The coupling terms shown in Eqs. S6 and S10 are not part of the standard Tavis-Cummings (TC) Hamiltonian.

The second term in Eq. (S1) gives rise to dipole self-energy (DSE) terms between ensemble states where the photonic excitation is not changing. These terms can be divided into three groups. The first group of terms provides state-specific energy shifts for the three types of molecular ensemble states:

$$\langle G;n|\,\tilde{\mu}^2\,|G;n\rangle = \sum_{a=1}^{N}\left(\mu_{gg}^2\right)^{(a)} + \mu_{gg}^{(a)}\left(\sum_{b\neq a}^{N}\mu_{gg}^{(b)} - 2\langle\hat{\mu}\rangle_0\right) + \langle\hat{\mu}\rangle_0^2 \tag{S11}$$

$$\begin{aligned}\big\langle E^{(a)};n\big|\,\tilde{\mu}^2\,\big|E^{(a)};n\big\rangle &= \left(\mu_{ee}^2\right)^{(a)} + \mu_{ee}^{(a)}\left(\sum_{b=1}^{N-1}\mu_{gg}^{(b)} - 2\langle\hat{\mu}\rangle_0\right) + \langle\hat{\mu}\rangle_0^2\\ &+\sum_{b=1}^{N-1}\left(\mu_{gg}^2\right)^{(b)} + \mu_{gg}^{(b)}\left(\sum_{c\neq b}^{N-1}\mu_{gg}^{(c)} - 2\langle\hat{\mu}\rangle_0\right)\end{aligned} \tag{S12}$$

$$\begin{aligned}\big\langle \mathcal{E}^{(a,b)};n\big|\,\tilde{\mu}^2\,\big|\mathcal{E}^{(a,b)};n\big\rangle &= \left(\mu_{ee}^2\right)^{(a)} + \left(\mu_{ee}^2\right)^{(b)} + 2\mu_{ee}^{(a)}\mu_{ee}^{(b)} + \langle\hat{\mu}\rangle_0^2\\ &+\left(\mu_{ee}^{(a)}+\mu_{ee}^{(b)}\right)\left(\sum_{c=1}^{N-2}\mu_{gg}^{(c)} - 2\langle\hat{\mu}\rangle_0\right)\\ &+\sum_{c=1}^{N-2}\left(\mu_{gg}^2\right)^{(c)} + \mu_{gg}^{(c)}\left(\sum_{d\neq c}^{N-2}\mu_{gg}^{(d)} - 2\langle\hat{\mu}\rangle_0\right)\end{aligned} \tag{S13}$$

The second group of DSE contributions couples state which share the same type of molecular

ensemble states.

$$\left\langle E^{(a)};n\right|\tilde{\mu}^2\left|E^{(b)};n\right\rangle = \mu_{eg}^{(a)}\mu_{eg}^{(b)} \tag{S14}$$

$$\left\langle \mathcal{E}^{(a,b)};n\right|\tilde{\mu}^2\left|\mathcal{E}^{(b,c)};n\right\rangle = \mu_{eg}^{(a)}\mu_{eg}^{(c)} \tag{S15}$$

$$\left\langle \mathcal{E}^{(a,b)};n\right|\tilde{\mu}^2\left|\mathcal{E}^{(c,d)};n\right\rangle = 0 \tag{S16}$$

The last group of DSE coupling connects states which share different types of molecular ensemble states.

$$\langle G;n|\,\tilde{\mu}^2\left|E^{(a)};n\right\rangle = \left(\mu_{eg}^2\right)^{(a)} + \mu_{eg}^{(a)}\left(\sum_{b=1}^{N-1}\mu_{gg}^{(b)} - 2\langle\hat{\mu}\rangle_0\right) \tag{S17}$$

$$\langle G;n|\,\tilde{\mu}^2\left|\mathcal{E}^{(a,b)};n\right\rangle = \mu_{eg}^{(a)}\mu_{eg}^{(b)} \tag{S18}$$

$$\left\langle E^{(a)};n\right|\tilde{\mu}^2\left|\mathcal{E}^{(a,b)};n\right\rangle = \left(\mu_{eg}^2\right)^{(b)} + \mu_{ee}^{(a)}\mu_{eg}^{(b)} + \mu_{eg}^{(b)}\left(\sum_{c=1}^{N-2}\mu_{gg}^{(c)} - 2\langle\hat{\mu}\rangle_0\right) \tag{S19}$$

$$\left\langle E^{(a)};n\right|\tilde{\mu}^2\left|\mathcal{E}^{(b,c)};n\right\rangle = 0 \tag{S20}$$

To reduce the complexity of the interaction Hamiltonian in the extended molecular Tavis-Cummings model, we apply the commonly used rotating wave approximation (RWA) [1]. This approximation affects all coupling terms whose contributions to the system dynamics are negligible. In the near-resonance regime, the liner dipole coupling terms of Eq. (S6) oscillate at twice the field frequency. Thus, the contribution of the coupling to the dynamics of the system is negligible. This argument does not hold for the coupling between states of the same matter excitation of Eq. (S10) arising from the permanent dipole moment component, since its time evolution in the interaction picture is driven by the optical field frequency. The squared transition dipole moment couplings of Eqs. (S17), (S18) and (S19) have a negligible influence on the system dynamics due to the large energy difference between the coupled states together with the squared coupling parameter λ^2.

To verify the RWA we determine the influence of the neglected terms on the population dynamics for a single MgH^+molecule coupled to a cavity. The extended molecular Tavis-Cummings (ETC) Hamiltonian of single molecule coupled to single cavity mode in the RWA reads:

$$\hat{H} = \hat{H}_M + \omega_c\left(\hat{a}^\dagger\hat{a} + \frac{1}{2}\right) - \sqrt{\frac{\omega_c}{2}}\lambda\mu_{eg}\left(\hat{a}\hat{\sigma}^\dagger + \hat{a}^\dagger\hat{\sigma}\right) - \sqrt{\frac{\omega_c}{2}}\lambda\left(\hat{a}+\hat{a}^\dagger\right)\left(\mu_{ee} - \langle\hat{\mu}\rangle_0\right)\hat{\sigma}^\dagger\hat{\sigma}$$
$$+ \frac{1}{2}\lambda^2\left(\mu_{gg}^2 - \langle\hat{\mu}\rangle_0^2\right)\hat{\sigma}\hat{\sigma}^\dagger + \frac{1}{2}\lambda^2\left(\mu_{ee}^2 - 2\mu_{ee}\langle\hat{\mu}\rangle_0 + \langle\hat{\mu}\rangle_0^2\right)\hat{\sigma}^\dagger\hat{\sigma}. \tag{S21}$$

The population dynamics of the $|g, 1\rangle$ state obtained using the standard molecular Jaynes-Cummings (JC) model, i.e. including only the transition dipole moment coupling, see Eq. (S7), is plotted in Fig. S1 a) and used as a reference in the following. If only the additional linear dipole coupling term is included, see Eq. (S6), the difference in population dynamics with respect to the molecular JC result is practically zero for the coupling strength used, see Fig. S1 b). In the case of a single molecule, only the DSE term shown in Eq. (S17) exists and is therefore affected by the RWA. Its influence on the $|g, 1\rangle$ dynamics with respect to the molecular JC population is very small, as shown in Fig. S1 c). To investigate the influence of the RWA on the ETC model, we compare the population difference with respect to the molecular JC result obtained with the RWA (purple curve in Fig. S1 d)) and without the RWA (pink curve in Fig. S1 d)). The small observed differences indicate that the RWA can be safely applied and that the contributions of Eqs. (S6), (S17), (S18) and (S19) can be neglected.

To understand the effects of the intermolecular dipole-dipole term arising from the DSE contribution of Eq. (S14), we propagated the Hamiltonians without its contribution. The differences in the propagation with respect to the molecular TC is shown in Fig. S2. The presence of this coupling in the ETC model increases the population difference in the case of $N = 3$ molecules.

In Fig. S3 we plot the variation in the population of state $|G, 1\rangle$ for the cases where the molecular TC model is extended with only the static dipole moment coupling or the DSE coupling terms. We note that the increase of molecules in our model changes the phase of the population difference of the static dipole moment contribution. In the case of $N = 2$ the near perfect matching of the contributions leads to a canceling of the overall effect when both coupling terms are considered (molecular ETC model).

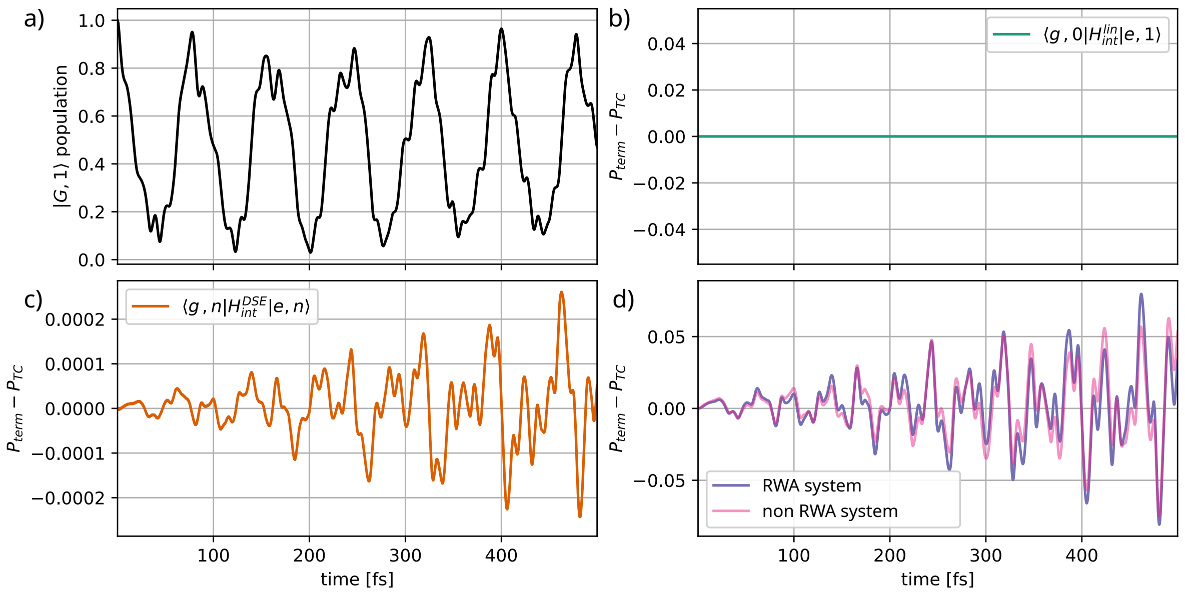

FIG. S1. a) Population dynamics of state $|g, 1\rangle$ for a single MgH^+molecule coupled to a cavity obtained using the standard molecular JC Hamiltonian. b) Difference in the population dynamics with respect to the JC results if only the counter-rotation coupling Eq. (S6) is included. c) Population difference if only the DSE coupling (see Eq. (S17)) is included. d) Population differences of the ETC dynamics with the RWA (purple) and without the RWA (pink). The cavity frequency is $4.322\,\mathrm{eV}$ and a coupling strength of 6.9×10^{-3} au is used.

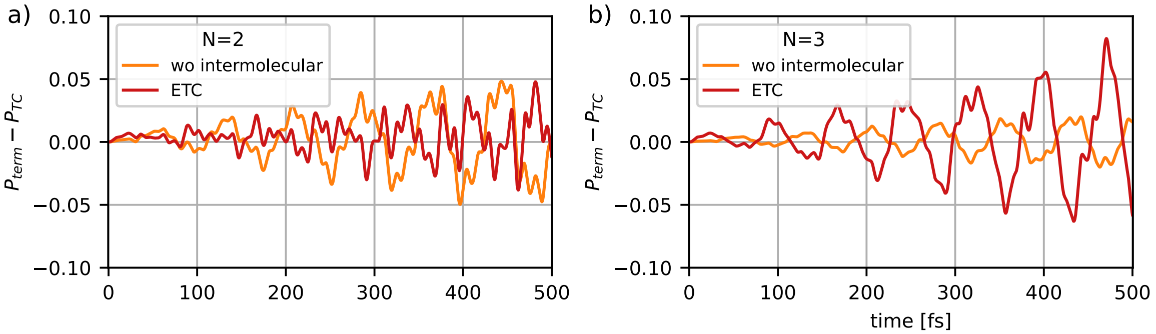

FIG. S2. Difference in the population dynamics of state $|G; 1\rangle$ with respect to the molecular TC model results for a) two and b) three molecules, considering the ETC model (red curve) and without the intermolecular dipole-dipole coupling (orange curves).

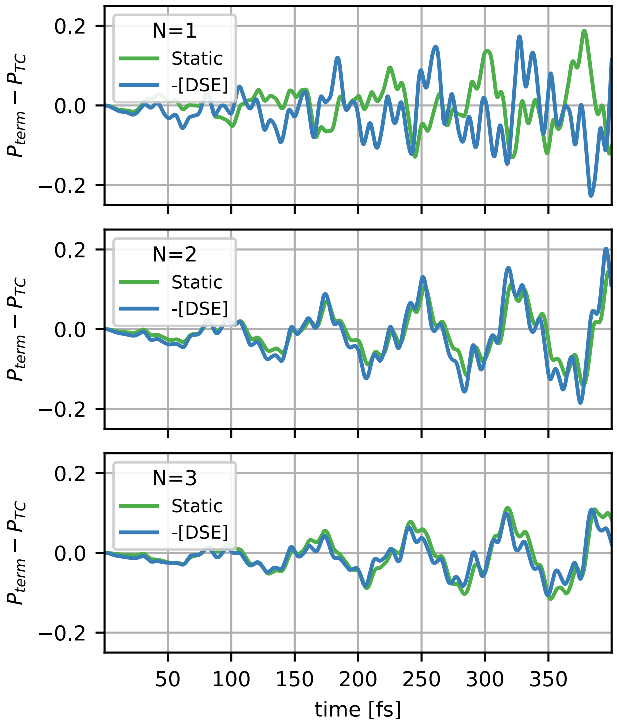

FIG. S3. Difference in the population dynamics of state $|G;1\rangle$ with respect to the molecular TC model results for one, two and three molecules, including the static dipole moment and DSE coupling terms separately in the molecular TC model Hamiltonian. The DSE population difference sign was inverted ($-[P_{\mathrm{DSE}} - P_{\mathrm{TC}}]$) to compare it with the static dipole moment contribution.

S2. ABSORPTION SPECTRA

The absorption spectra for the coupled molecular-cavity system were calculated by Fourier transform of the time-dependent expectation value of the total dipole moment. The resulting absorption spectra contain both vibrational and electronic transitions in the coupled molecule-cavity systems. In Fig. S4 these spectral regions are shown for one, two, and three MgH^+molecules coupled to a cavity (black) and without cavity coupling (orange). The transition between the lower polariton (LP) state and the upper polariton (UP) state is the most prominent feature in the IR spectra, left column of Fig. S4. The observed Rabi frequency Ω_R of $52.1\,meV$, for the single molecule case, is slightly redshifted compared to the approximate value of $\sqrt{2\omega_c}\lambda\mu_{eg} = 57\,meV$. The UV spectra (right column of Fig. S4) show the formation of the LP and UP states, which are strongly red-shifted with respect to the cavity frequency ω_c. Within the vibrational progression, peaks are shifted, and additional splittings due to the light-matter interaction are visible, e.g. around 4.7 eV.

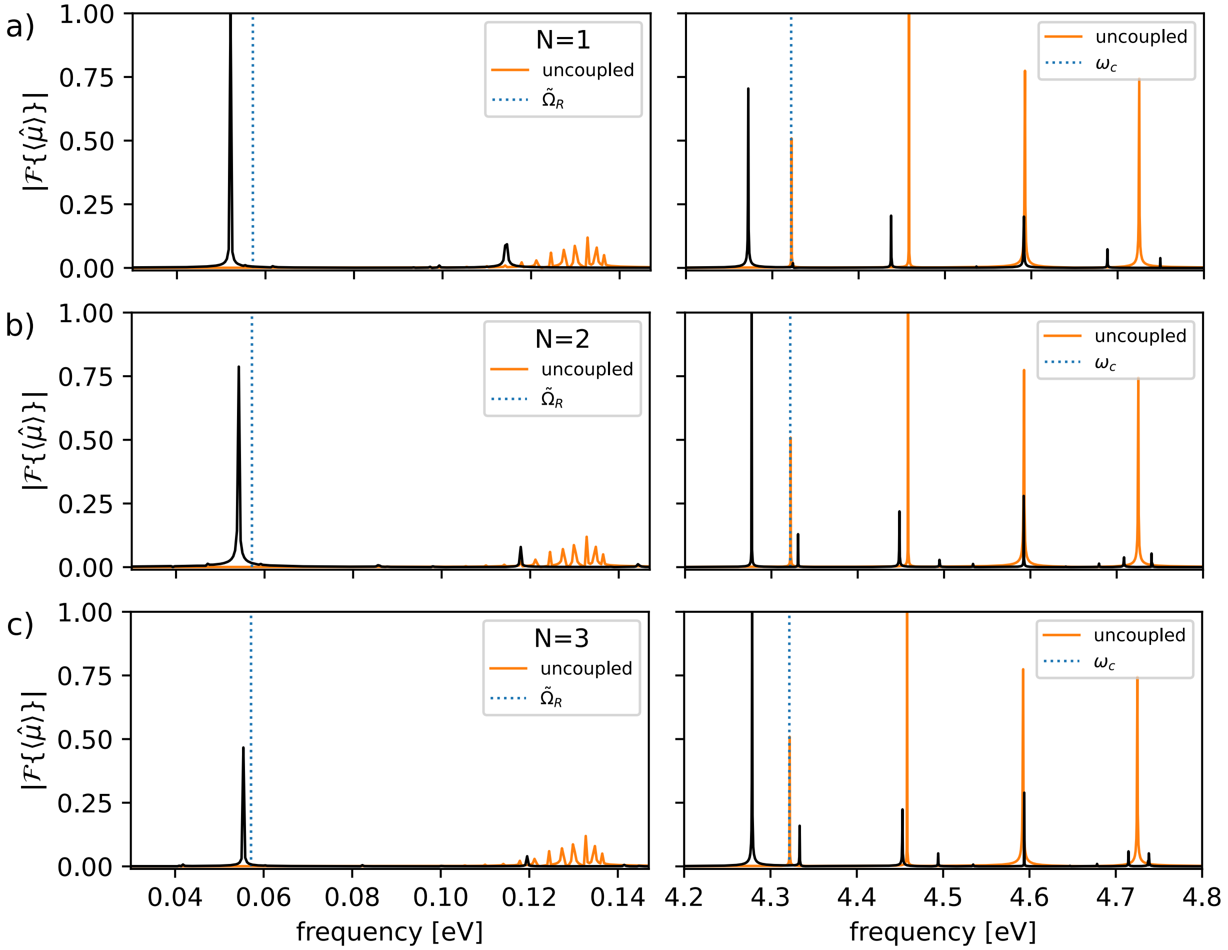

FIG. S4. Polaritonic absorption spectra for a) one, b) two and c) three molecule considering the ETC model with coupling strength $\sqrt{N}\lambda_c = 6.9 \times 10^{-3}$ au and cavity excitation frequency $\omega_c = 4.322$ eV (black curve). The orange curve corresponds to the uncoupled case. The left column shows the IR part of the spectra, the right column shows the relevant part of the UV spectra. The blue dotted lines in the left column correspond to the Rabi frequency of the equivalent TLS model $\tilde{\Omega}_R$, and in the right column, they are indicating the cavity frequency ω_c.

S3. POLARITONIC FRANCK-CONDON FACTORS

The observed red shifts in the absorption spectra of the coupled MgH^+-cavity system can be explained by the interplay of the inherent anharmonicity of the potential energy surfaces (PESs) and the geometrical displacement δb between the two PESs. To investigate how large these two contributions are, we constructed a simplified TC model that contains two electronic states, each with 10 vibrational states. To unravel the two effects, we used three different pairs of PESs to describe the two electronic states shown in Fig. S5 and Fig. S8: a) two identical harmonic potentials, b) two Morse potentials with different dissociation energies, and c) the two real molecular PESs. In the three cases shown in Fig. S6 and Fig.S8, we vary the geometric displacement δb, determine the transition dipole moments factors (using the molecular transition dipole moment function) for all pairs of vibrational states between the two electronic states, and couple the resulting 20-level system to a single cavity mode. The resulting first four to five polaritonic states as a function of δb are plotted in Figs. S6 and S8. To monitor the asymmetry of the Rabi splitting, we calculate the average energy $\overline{\omega}_P$ of the LP and UP eigenstates. If $\overline{\omega}_P$ is identical to the uncoupled eigenstates (forming LP and UP), the Rabi splitting is perfectly symmetric if its smaller or larger, LP and UP are redshifted or blueshifted, respectively. Even in the case of identical harmonic potentials (Fig. S6 a)) the Rabi splitting is symmetric only if $\delta b = 0$ otherwise a weak red shift is observed due to the nonzero contribution from higher vibrational states. The inherent anharmonicity of the Morse potentials only slightly increases the redshift of the Rabi splitting, see Fig. S6 b).

For the molecular case, we observe a significantly larger redshift of the average polariton frequency for $\delta b \neq 0$ compared to the two model systems, see Fig. S8 a). As the potentials are softer and more asymmetric/anharmonic, the vibrational levels are closer in energy. And as a consequence, the influence of higher states on the LP and UP state is significantly stronger.

As a benchmark, we compare the absorption spectra peaks for the coupled single molecule system with the eigenvalues of the coupled vibrational multilevel states system in Fig. S9, reaching a satisfactory agreement between curves with different vacuum field coupling strengths.

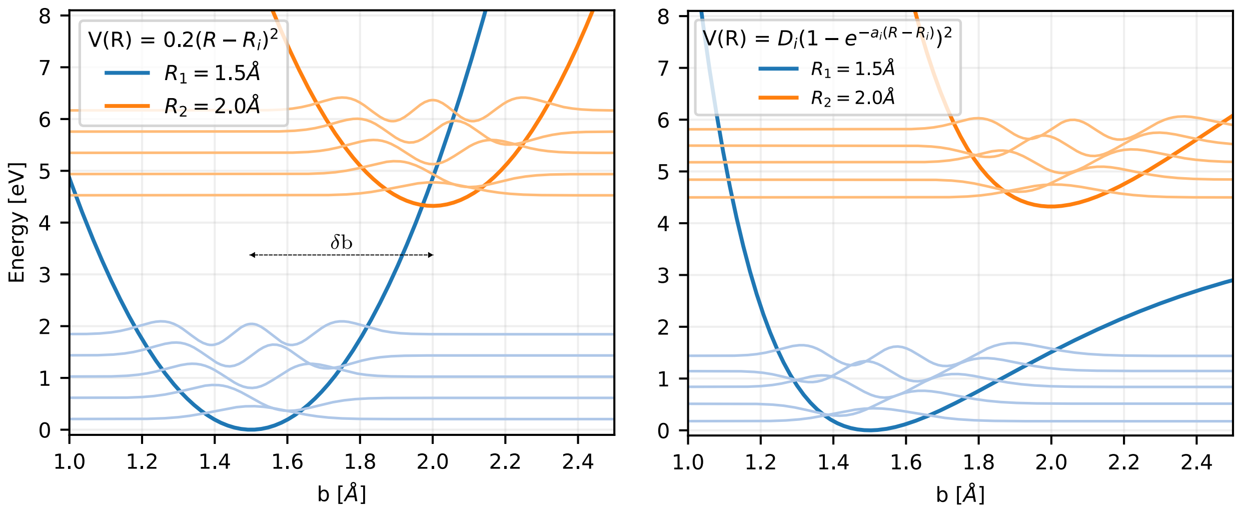

FIG. S5. Sketch of the electronic potentials (dark curves) and respective vibrational eigenstates (light curves) shifted by their eigenvalues. a) Harmonic potentials are given by a parabolic equation. b) Morse potentials with dissociation energies $D_1 = 4$eV and $D_2 = 6$eV, where $\mathrm{a}_i = \sqrt{0.15/D_i}$.

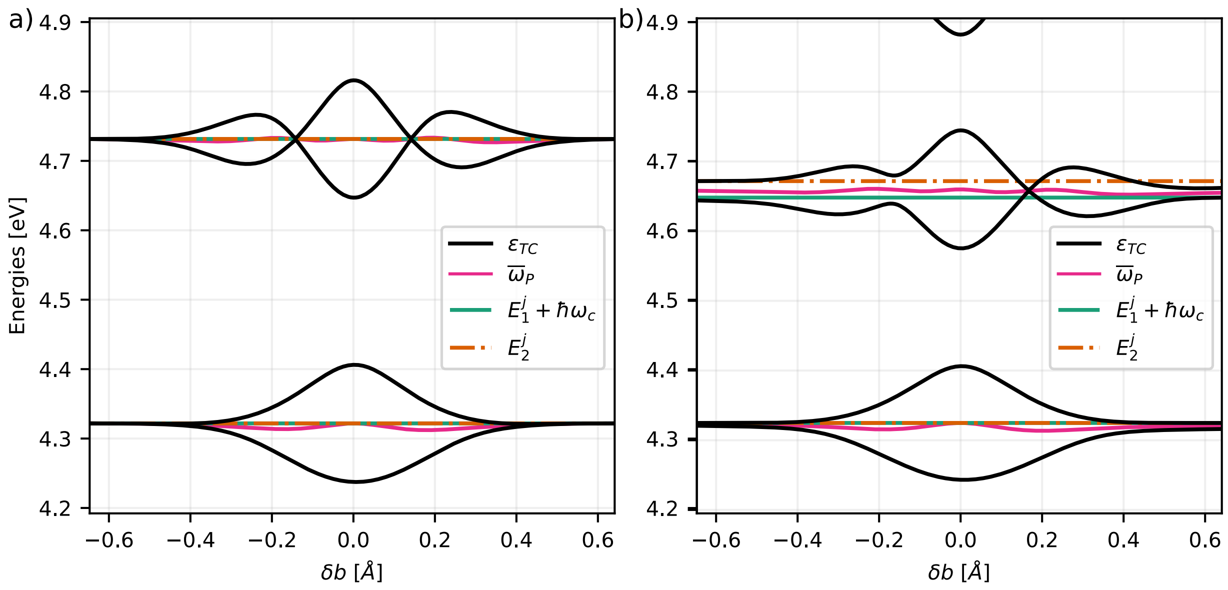

FIG. S6. Polaritonic eigenenergies for the multilevel systems with a) equal harmonic potentials and b) different Morse potentials with dissociation energies of $D_1 = 2$ eV and $D_2 = 8$ eV.

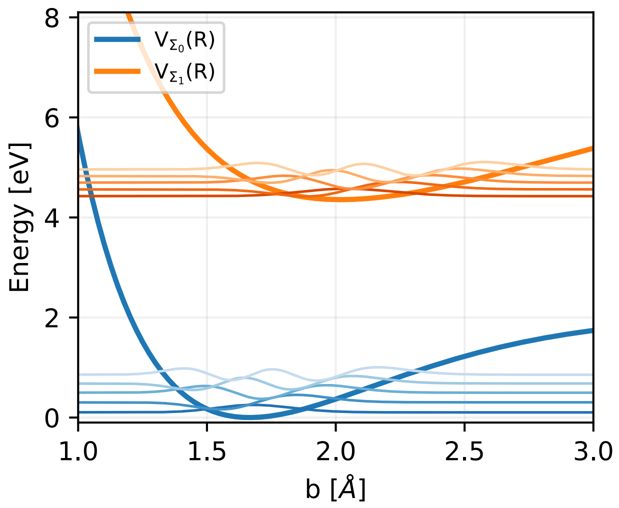

FIG. S7. Sketch of the MgH^+electronic potentials (dark curves) and respective vibrational eigenstates (light curves) shifted by their eigenvalues.

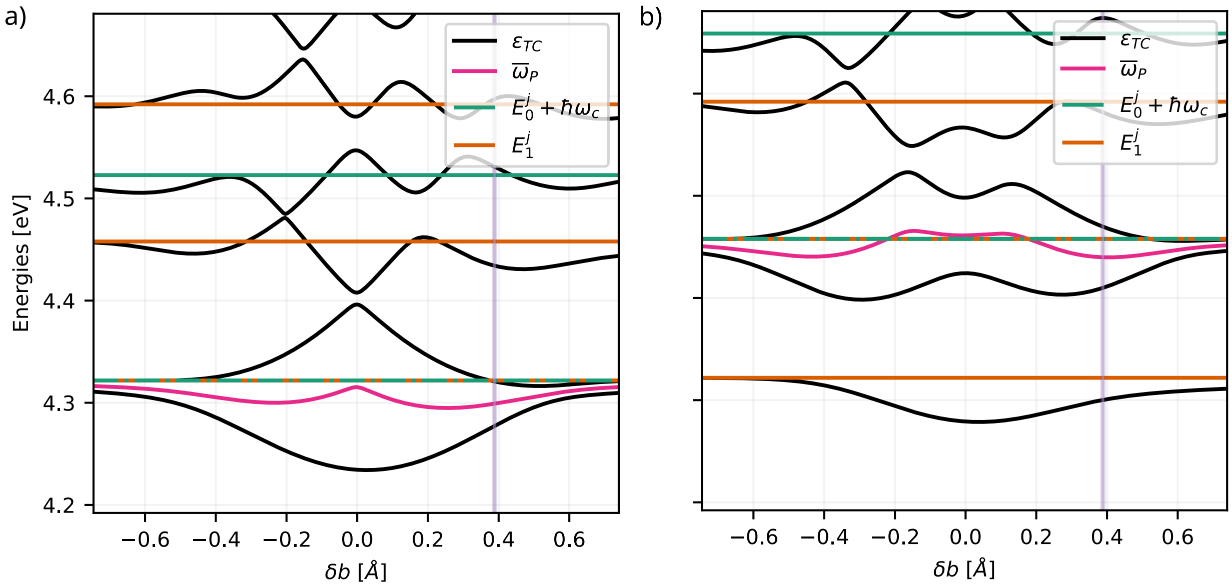

FIG. S8. Polaritonic eigenenergies for the multilevel systems with molecular potentials resonant a) with the $|g, v = 0\rangle \to |e, v = 0\rangle$ transition and b) at with the $|g, v = 0\rangle \to |e, v = 1\rangle$ transition. Purple line denotes the natural relative position between the potentials.

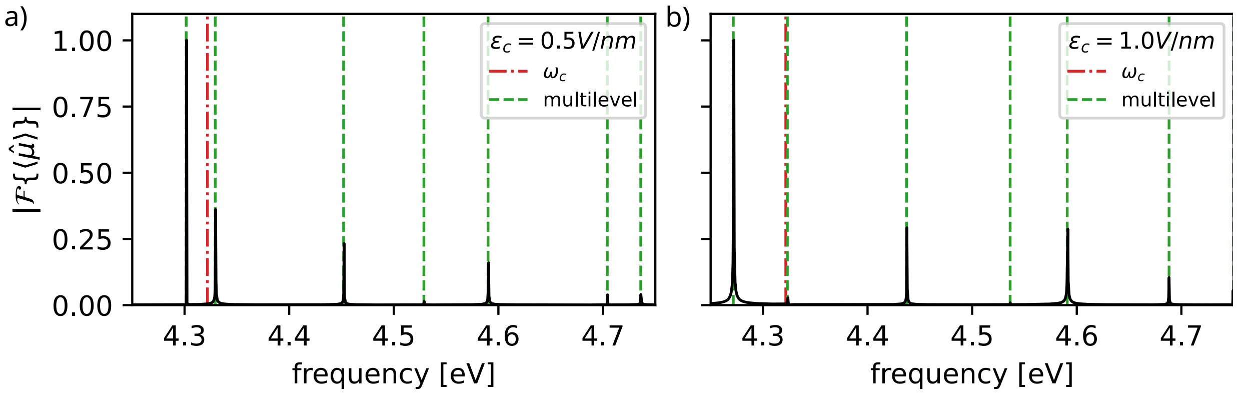

FIG. S9. Absorption spectra for the molecular propagations of the ETC model, compared to the multilevel eigenvalues (green dotted lines). Propagations and multilevel systems considering a cavity frequency of $\omega_c = 4.322$ eV and coupling strengths of a) 3.5×10^{-3} au and b) 6.9×10^{-3} au.

S4. DERIVATION OF THE EFFECTIVE MOLECULAR TAVIS-CUMMINGS MODEL

To reduce computational costs, the molecules coupled to the cavity mode are replaced by effective TLSs. The two levels represent the first vibrational eigenstate of the two electronic states $|g\rangle$ and $|e\rangle$, separated by ω_{eg}. The individual molecular Hamiltonian reduces to

$$H_M = \omega_{eg}\hat{\sigma}^\dagger\hat{\sigma} \tag{S22}$$

The $\mathbf{R}$ dependent dipole matrix elements in eq. (S2) and the dipole moment squared functions in eq. (S5) are eplaced by the corresponding expectation values:

$$\mu_{ij}(\mathbf{R}) \to \langle\mu\rangle_{ij} = \left\langle\chi_i^0(\mathbf{R})\right|\mu_{ij}(\mathbf{R})\left|\chi_j^0(\mathbf{R})\right\rangle_{\mathbf{R}}, \tag{S23}$$

$$\mu_{ij}^2(\mathbf{R}) \to \left\langle\mu^2\right\rangle_{ij} = \left\langle\chi_i^0(\mathbf{R})\right|\mu_{ij}^2(\mathbf{R})\left|\chi_j^0(\mathbf{R})\right\rangle_{\mathbf{R}}. \tag{S24}$$

where $\chi_i^0(\mathbf{R})$ is the first vibrational eigenfunction of the electronic state i. In the coherent state basis, the expectation value of the total dipole moment and its quadratic counterpart for a system of N-TLSs have the form

$$\langle\tilde{\mu}\rangle = \sum_i^N \langle\mu\rangle^{(i)} - N\langle\mu\rangle_{gg}, \tag{S25}$$

$$\left\langle\tilde{\mu}^2\right\rangle = \sum_i^N \left\langle\mu^2\right\rangle^{(i)} - 2\langle\mu\rangle^{(i)} N\langle\mu\rangle_{gg} + \sum_{j\neq i}^N \langle\mu\rangle^{(j)}\langle\mu\rangle^{(g)} + N^2\langle\mu\rangle_{gg}^2, \tag{S26}$$

where i and j are the individual contributions from N TLSs. Since all TLSs are identical and independent of $\mathbf{R}$, the upper index of the dipole and squared dipole moments used to distinguish the individual molecules are dropped below.

The linear dipole moment couplings within the RWA take the following form in the TLS model:

$$\langle G;1|\left(\hat{a}^\dagger+\hat{a}\right)\langle\tilde{\mu}\rangle\left|E^{(a)};0\right\rangle = \langle\mu\rangle_{eg} \tag{S27}$$

$$\langle G;2|\left(\hat{a}^\dagger+\hat{a}\right)\langle\tilde{\mu}\rangle\left|E^{(a)};1\right\rangle = \langle\mu\rangle_{eg} \tag{S28}$$

$$\left\langle E^{(a)};1\right|\left(\hat{a}^\dagger+\hat{a}\right)\langle\tilde{\mu}\rangle\left|\mathcal{E}^{(a,b)};0\right\rangle = \langle\mu\rangle_{eg} \tag{S29}$$

$$\left\langle E^{(a)};1\right|\left(\hat{a}^\dagger+\hat{a}\right)\langle\tilde{\mu}\rangle\left|E^{(a)};0\right\rangle = \langle\mu\rangle_{ee} - \langle\mu\rangle_{gg} \tag{S30}$$

(S31)

The diagonal DSE contribution in the TLS model reads:

$$\langle G;n|\left\langle\tilde{\mu}^2\right\rangle|G;n\rangle = N\left\langle\mu^2\right\rangle_{gg} - N\left\langle\mu\right\rangle_{gg}^2 \tag{S32}$$

$$\left\langle E^{(a)};n\right|\left\langle\tilde{\mu}^2\right\rangle\left|E^{(a)};n\right\rangle = (N-1)\left\langle\mu^2\right\rangle_{gg} + \left\langle\mu^2\right\rangle_{ee} - (N-1)\left\langle\mu\right\rangle_{ee}\left\langle\mu\right\rangle_{gg} + \left\langle\mu\right\rangle_{gg}^2 \tag{S33}$$

$$\left\langle \mathcal{E}^{(a,b)};n\right|\left\langle\tilde{\mu}^2\right\rangle\left|\mathcal{E}^{(a,b)};n\right\rangle = (N-2)\left\langle\mu^2\right\rangle_{gg} + 2\left\langle\mu^2\right\rangle_{ee} + 2\left\langle\mu\right\rangle_{ee}^2 - 2(N-2)\left\langle\mu\right\rangle_{ee}\left\langle\mu\right\rangle_{gg} + 4\left\langle\mu\right\rangle_{gg}^2 \tag{S34}$$

The DSE contributions coupling state which share the same type of TLS ensemble states read:

$$\left\langle E^{(a)};n\right|\left\langle\tilde{\mu}^2\right\rangle\left|E^{(b)};n\right\rangle = \left\langle\mu\right\rangle_{eg}^2 \tag{S35}$$

$$\left\langle \mathcal{E}^{(a,b)};n\right|\left\langle\tilde{\mu}^2\right\rangle\left|\mathcal{E}^{(b,c)};n\right\rangle = \left\langle\mu\right\rangle_{eg}^2 \tag{S36}$$

Adding more TLSs to the system results in more coupling terms in the interaction Hamiltonian. However, due to the scaling of the coupling strength, the energy shift of the diagonal DSE contributions do not increase with the system size; see Fig. S10. The DSE ground state shift, given by Eq. (S32), (blue line in Fig. S10) remains constant, while the other two state-specific shifts, given by Eqs. (S33) and (S34) (green and orange lines), decay with one over N. However, they converge to a nonzero value. The DSE contributions coupling state which share the same type of TLS ensemble states, see Eqs. (S35) and (S36), are very small and decay to zero with one over N.

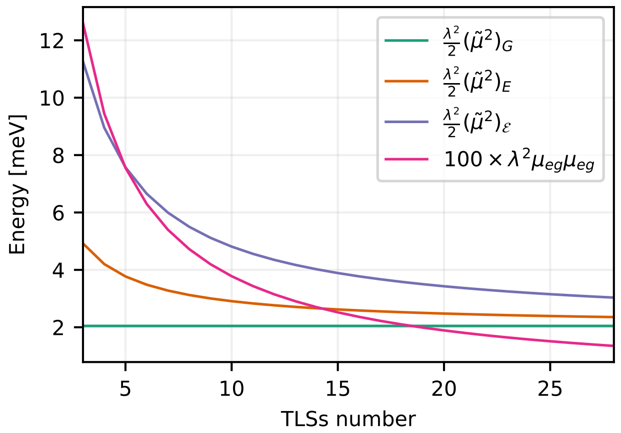

FIG. S10. Energy contributions of the diagonal DSE terms considered as functions of system size N. Coupling strength $\sqrt{N}\lambda_c = 6.9 \times 10^{-3}$ au and cavity frequency $\omega_c = 4.322$ eV.

Due to the simplicity of the TLS, we have analytical formulas to describe the population dynamics of its states and absorption spectrum. The Rabi frequency for the first manifold excitation states, which describes the oscillations between ground and excited states populations, is given by

$$\Omega_R = \sqrt{2N\omega_c(\lambda_c\mu_{eg})^2 + (\Delta\omega_c)^2}, \tag{S37}$$

where $\Delta\omega_c = \omega_{eg} - \omega_c$ is the cavity detuning to the TLS excitation frequency and N is the number of emitters. The polariton states eigenenergies are given by

$$E_\pm = \frac{(\omega_c + \omega_{eg})}{2} \pm \frac{\Omega_R}{2}, \tag{S38}$$

where $|UP\rangle \equiv |+\rangle$ and $|LP\rangle \equiv |-\rangle$.

The absorption spectra for a TLS can be calculated analogously to the molecular system case, and an example dispersion curve for the TLS model is shown in Fig. S11. The polariton state peaks follow hyperbolic curves whose asymptotes are the TLS excitation frequency ω_{eg}^{TLS} and the cavity frequency ω_c^{TLS}, centered at the resonance point.

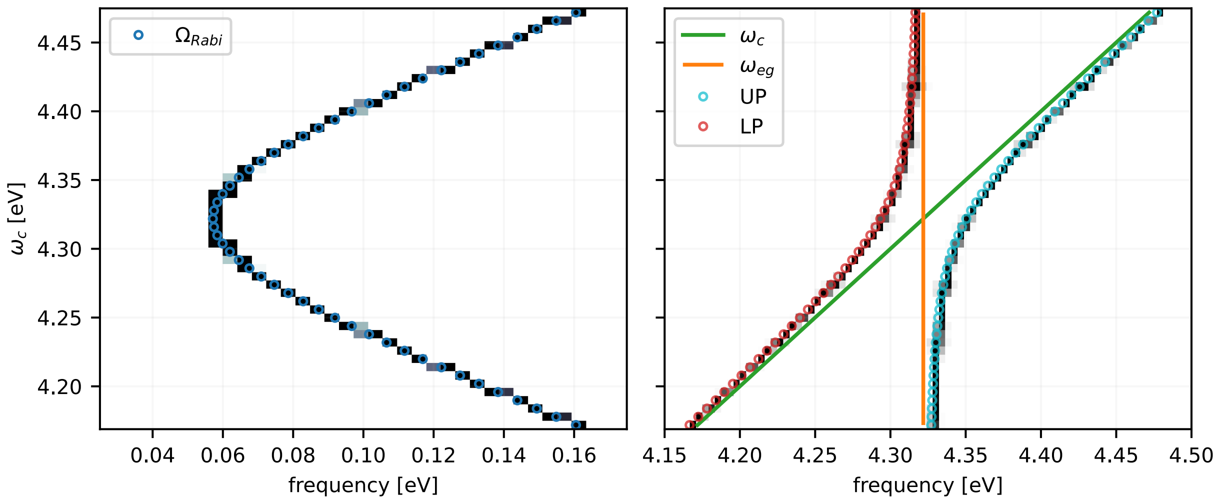

FIG. S11. Absorption spectrum for a 2-TLS system with respect to cavity frequency ω_c using a $\omega_{eg}^{TLS} = 4.322$eV and a coupling strength of $\sqrt{N}\lambda_c = 6.9 \times 10^{-3}$ au. Left panel shows the Rabi frequencies compared to Eq. (S37), and right panel shows the electronic transitions for the UP and LP states, compared to Eq. (S38).

By definition, the TLSs model is characterized by a symmetric Rabi splitting, see Fig. S11, and cannot reproduce the observed asymmetry in the polaritonic eigenstates; see Section S2.

Based on the eigenvalues of the coupled molecule-cavity system, it is possible to optimize the TLS to mimic the energetics of the real molecular polariton states. Starting from the dispersion curve of the molecule-cavity system, see Fig. S12 for the cases of one and two MgH^+, we define the region where $\overline{\omega}_P$ (pink line) is approximately linear. In the next step, we determine the energy gap between the two levels ω_{eg}^{TLS} and an effective detuning of the cavity frequency Δ_c^{TLS} with respect to the one used for the molecular setup. By defining a suitable $\overline{\omega}_P^{TLS}$ (pink dashed line Fig. S12), we can compute the calculated ω_{eg}^{TLS} and $\omega_g^{TLS}+\omega_c$ as the vertical asymptote (orange dashed line) and the diagonal asymptote (green dashed line), respectively. Furthermore, we also defined an optimal coupling strength λ_c^{TLS} from the Rabi frequency peak in the absorption spectra to replicate the molecular results.

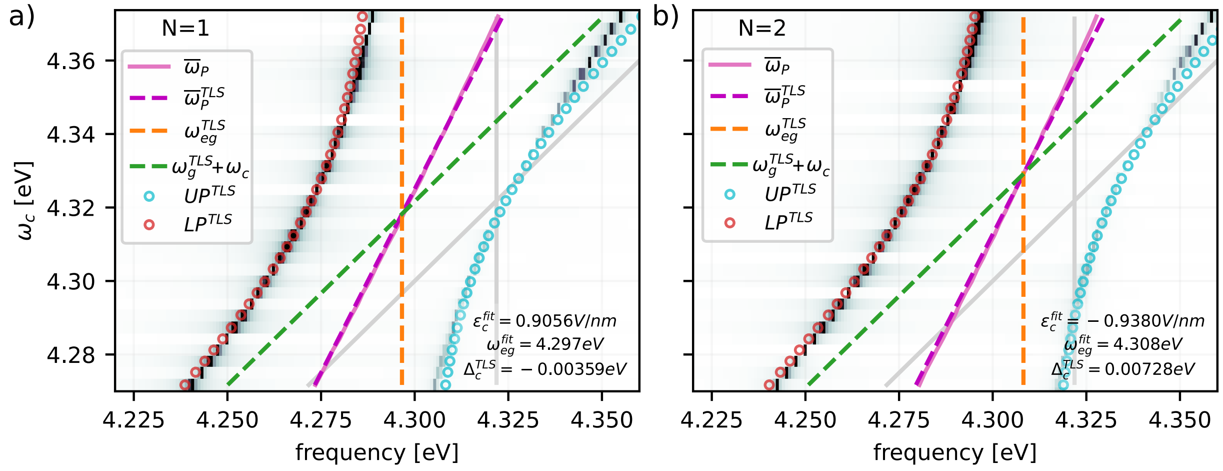

FIG. S12. Fitting of TLSs upper and lower polariton curves using the molecular dispersion curve for the case of a) one and b) two MgH^+molecules in the region where the middle polariton frequency $\overline{\omega}_P$ is almost linear. The fitted TLS polariton curves have asymptotes that correspond to the optimized emitter resonance energy ω_{eg}^{TLS}, and to the ground level energy ω_g^{TLS} plus cavity excitation which gives the detuning Δ_c^{TLS}. The gray lines correspond to the molecular parameters of ω_{eg} and $\omega_g+\omega_c$.

All optimized TLS parameters for one, two, and three MgH^+coupled to a cavity are listed in table S1. We use the optimized parameter of the three-molecule case as default for larger ensembles of TLSs considering that the resonance frequency seems to converge in the molecular case as the system grows.

The curves of Fig. 10 and Fig. 11(a) were fitted to the formula $f(N) = a + bN^c$ using the nonlinear least squares method. The covariance matrices for the fitted curves of Max. ΔP,

TABLE S1. Optimization parameters for the TLSs fitted in the molecular absorption spectra used in the main text.

N	1	2	3
λ_c^{TLS} [10^{-3}au]	6.25	6.47	6.52
ω_{eg}^{TLS} [eV]	4.297	4.308	4.312
Δ_c^{TLS} [meV]	-3.590	7.280	11.60

ΔE_{LP}, ΔE_{D} and ΔE_{UP} are given in the following equations, respectively, where the square root of the diagonals gives the standard deviation errors on the parameters a, b and c.

$$\begin{pmatrix} 2.111\,600\times10^{-10} & 1.509\,237\times10^{-9} & -1.059\,977\times10^{-8} \\ 1.509\,237\times10^{-9} & 1.600\,748\times10^{-8} & -9.234\,544\times10^{-8} \\ -1.059\,977\times10^{-8} & -9.234\,544\times10^{-8} & 5.918\,056\times10^{-7} \end{pmatrix}, \tag{S39}$$

$$\begin{pmatrix} 9.854\,209\times10^{-16} & 7.630\,624\times10^{-15} & -5.157\,231\times10^{-12} \\ 7.630\,624\times10^{-15} & 8.785\,710\times10^{-14} & -4.895\,806\times10^{-11} \\ -5.157\,231\times10^{-12} & -4.895\,806\times10^{-11} & 3.019\,670\times10^{-8} \end{pmatrix}, \tag{S40}$$

$$\begin{pmatrix} 5.409\,534\times10^{-17} & 4.268\,03\times10^{-16} & -1.725\,555\times10^{-13} \\ 4.268\,043\times10^{-16} & 5.009\,693\times10^{-15} & -1.671\,352\times10^{-12} \\ -1.725\,555\times10^{-13} & -1.671\,352\times10^{-12} & 6.166\,764\times10^{-10} \end{pmatrix}, \tag{S41}$$

$$\begin{pmatrix} 1.397\,314\times10^{-15} & 1.134\,212\times10^{-14} & -1.142\,411\times10^{-11} \\ 1.134\,212\times10^{-14} & 1.370\,908\times10^{-13} & -1.140\,863\times10^{-10} \\ -1.142\,411\times10^{-11} & -1.140\,863\times10^{-10} & 1.048\,726\times10^{-7} \end{pmatrix}. \tag{S42}$$

S5. RESOLUTION OF IDENTITY APPROACH FOR THE SQUARED DIPOLE OPERATORS

The squared dipole operators used in the DSE coupling terms were calculated by the resolution of the identity approach given in Eq. 18. The convergence of the summation is shown in Fig. S13, where $\mathcal{S}_m$ refers to the partial summation on the electronic states of $\langle\hat{\mu}^2\rangle_{kl} = \sum_{i=0}^{m} \mu_{ki}\mu_{il}$. As can be seen from the plots, the convergence of the summations are satisfactory using only the first five electronic states.

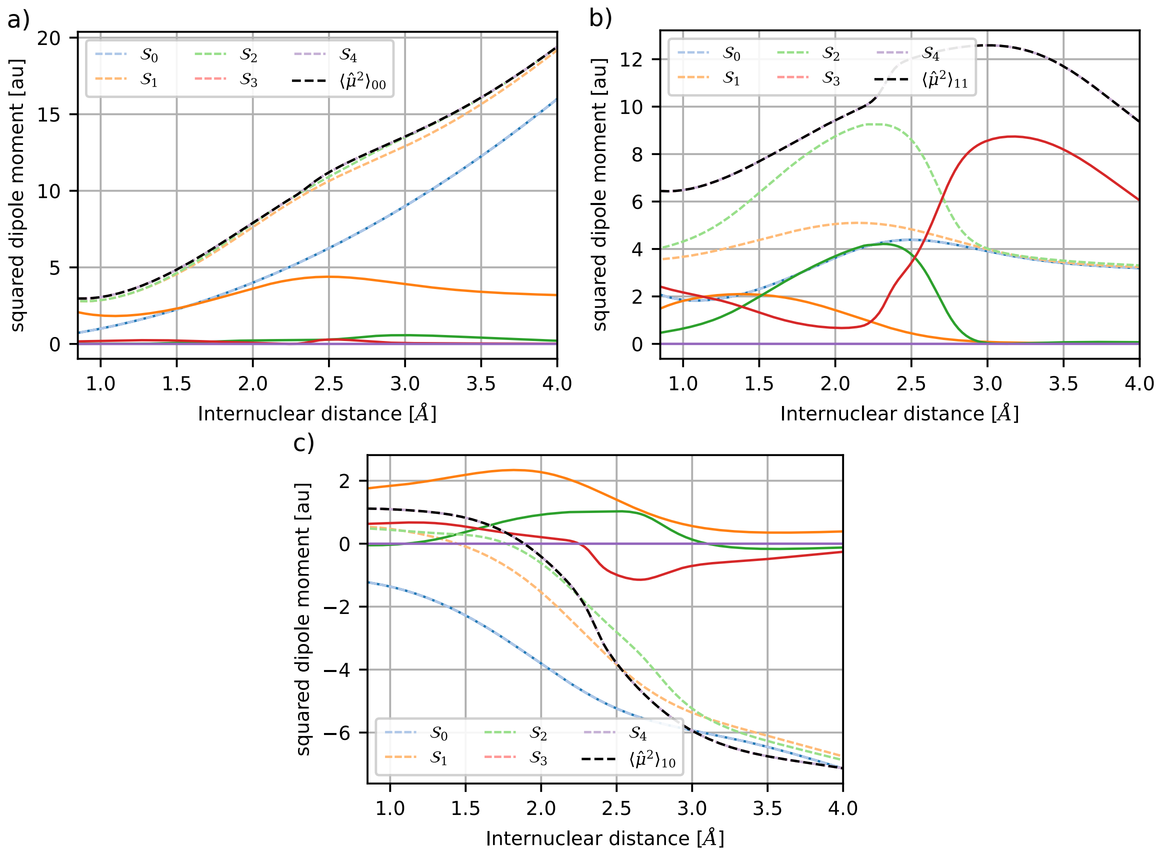

FIG. S13. Resolution of identity for the squared dipole moments of a) $\mu_{gg}^2 = \langle\hat{\mu}^2\rangle_{00}$, b) $\mu_{ee}^2 = \langle\hat{\mu}^2\rangle_{11}$ and c) $\mu_{eg}^2 = \langle\hat{\mu}^2\rangle_{10}$, where $\mathcal{S}_m$ is given by $\Sigma_i^m \mu_{0i}\mu_{i0}$, $\Sigma_i^m \mu_{1i}\mu_{i1}$ and $\Sigma_i^m \mu_{1i}\mu_{i0}$, respectively, and darker curves correspond to the final terms of the partial summations.

[1] "Atom-field interaction," in *Quantum Optics in Phase Space* (John Wiley & Sons, Ltd, 2001) Chap. 14, pp. 381–412.
